# Study on the Structure-Activity Relationship of an Antimicrobial Peptide, Brevinin-2GUb, from the Skin Secretion of *Hylarana guentheri*

**DOI:** 10.3390/antibiotics10080895

**Published:** 2021-07-22

**Authors:** Yaxian Lin, Siyan Liu, Xinping Xi, Chengbang Ma, Lei Wang, Xiaoling Chen, Zhanzhong Shi, Tianbao Chen, Chris Shaw, Mei Zhou

**Affiliations:** 1 School of Pharmacy, Queen’s University Belfast, 97 Lisburn Road, Belfast BT9 7BL, UK; ylin36@qub.ac.uk (Y.L.); sliu25@qub.ac.uk (S.L.); c.ma@qub.ac.uk (C.M.); x.chen@qub.ac.uk (X.C.); t.chen@qub.ac.uk (T.C.); chris.shaw@qub.ac.uk (C.S.); m.zhou@qub.ac.uk (M.Z.); 2 Department of Natural Sciences, Faculty of Science and Technology, Middlesex University, London NW4 4BT, UK; z.shi@mdx.ac.uk

**Keywords:** antimicrobial peptides, Brevinin-2GUb, structure–activity relationship

## Abstract

Antimicrobial peptides (AMPs) are considered potential alternatives to antibiotics due to their advantages in solving antibiotic resistance. Brevinin-2GUb, which was extracted from the skin secretion of *Hylarana guentheri*, is a peptide with modest antimicrobial activity. Several analogues were designed to explore the structure–activity relationship and enhance its activity. In general, the Rana box is not an indispensable motif for the bioactivity of Brevinin-2GUb, and the first to the 19th amino acids at the N-terminal end are active fragments, such that shortening the peptide while maintaining its bioactivity is a promising strategy for the optimisation of peptides. Keeping a complete hydrophobic face and increasing the net charges are key factors for antimicrobial activity. With the increase of cationic charges, α-helical proportion, and amphipathicity, the activity of t-Brevinin-2GUb-6K (tB2U-6K), in combatting bacteria, drastically improved, especially against Gram-negative bacteria, and the peptide attained the capacity to kill clinical isolates and fungi as well, which made it possible to address some aspects of antibiotic resistance. Thus, peptide tB2U-6K, with potent antimicrobial activity against antibiotic-resistant bacteria, the capacity to inhibit the growth of biofilm, and low toxicity against normal cells, is of value to be further developed into an antimicrobial agent.

## 1. Introduction

Antimicrobial resistance has reached alarming levels in many parts of the world, and is considered one of the most significant public health threats [1]. The rise of antimicrobial resistance has led to more treatment failures, spread broader infections in healthcare institutions and communities, and conferred a heavier economic burden on governments [1]. Therefore, it is critical to find new antimicrobial agents to deal with this problem.

AMPs have been used as potential alternatives for antibiotics; they have improved our living environments, but have become ineffective due to the emergence of multidrug-resistance microorganisms [2]. AMPs play an important and diverse role in the innate immune system, which is the first primary defence against pathogens [3]. They exhibit rapid and broad-spectrum antimicrobial activity against Gram-negative and Gram-positive bacteria and fungi, and some AMPs even have the capacity to combat antibiotic-resistant bacterial strains [4]. Conventional antibiotics kill bacteria through specific targets, such as penicillin, which binds with the transpeptidase on the bacteria to inhibit cell wall formation [5]. Most AMPs, differing from traditional antibiotics, have no discrete targets, but instead, they attach to the bacterial membrane through electrostatic interaction and kill the pathogens via membrane disruption, acting via various models [6], which make the pathogens more challenged to become resistant to AMPs [2].

There are some common structural characteristics for AMPs, including charge, conformation, hydrophobicity, and amphipathicity. All of the bacterial membranes represent an anionic surface [7], so that most antibacterial peptides contain basic amino acids that provide positive charges, such as lysine or arginine. Then, the peptides can adsorb to the bacterial membrane through electrostatic interaction, which is the prerequisite for disrupting the membrane. There is a strong correlation between charge and antimicrobial activity [8]. More positive charges will result in greater antimicrobial activity within a certain range, but if the number of the charges is over the critical threshold, the haemolytic toxicity will be increased [9]. Most AMPs share a common characteristic: amphiphilic construction, which means they possess hydrophobic and hydrophilic properties, and it is an important feature to differentiate between antimicrobial peptides with or without anti-Gram-negative bacteria activities, and the peptide with higher amphipathicity tends to possess dual activities against bacteria and fungi [10].

Based on the similarities in the structural characteristics and the capacity to combat pathogens, the frog skin secretion-derived antimicrobial peptides are grouped into different peptide families, such as brevinins, temporins, magainins, etc. [11]. The peptides in the brevinin family, isolated from the Ranidae, are poorly conserved in their primary structure. However, they contain a highly-conserved motif, called a ‘Rana box’. A Rana box is a disulphide bridge formed by two flanking cysteine residues with the second being at the C-terminus, and four or five amino acids located in the middle [12]. The antimicrobial activities of brevinin peptides are strong and broad-spectrum. The peptides in the brevinin-2 subfamily have much weaker haemolytic activity than the peptides in the brevinin-1 subfamily and the peptides were reported to possess other functions, playing an essential role in the innate immune system, such as anti-inflammatory activity [13]. Brevinin-2GUb, which was extracted from the skin secretion of *Hylarana guentheri*, was proven to have antimicrobial activity with low cytotoxicity [14].

Although AMPs have received attention as effective antimicrobial agents because of these advantages, there are still several limitations inhibiting the development of their clinical use. Firstly, the cationic residues on the antimicrobial peptides force them to bind to the bacterial membrane selectively through electrostatic interaction. However, since the antimicrobial peptides always contain similar functions to eukaryotic nuclear localisation signal peptides, they can be transferred into cells and induce mast cell degranulation, resulting in cytotoxicity [2]. Secondly, AMPs are susceptible to the features of their surrounding environment, including physiological salt concentration, pH, and serum conditions [15]. Moreover, antimicrobial peptides can be rapidly degraded by proteolytic enzymes from the digestive system or blood plasma, which will cause a low oral bioavailability of the peptides [15,16]. Finally, the high production cost is one of the principal limitations for utilising AMPs as potential clinical drugs [17]. Therefore, these obstacles need to be overcome to improve the chances of antimicrobial peptides to become clinical drugs.

In this article, several analogues of Brevinin-2GUb were designed to explore the structure–activity relationship and further enhance antimicrobial activity. These peptides were synthesised, and the purified samples were utilised for a series of bioassays. After these analyses, the antimicrobial activities of Brevinin-2GUb, t-Brevinin-2GUb-K (tB2U-K) and tB2U-6K against *Escherichia coli* (*E. coli),* were verified in the wax moth infection model.

## 2. Results

### 2.1. Peptide Design

**Figure 1:**
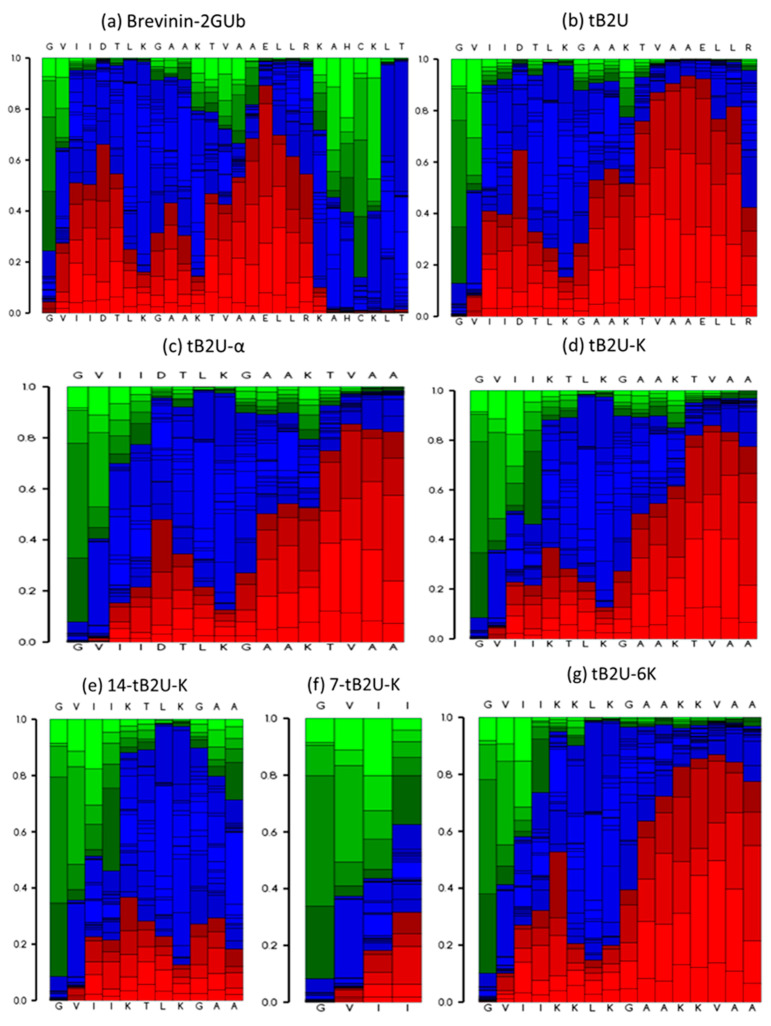
The secondary structure of (**a**) Brevinin-2GUb, (**b**) tB2U, (**c**) tB2U-α, (**d**) tB2U-K, (**e**) 14-tB2U-K, (**f**) 7-tB2U-K, and (**g**) tB2U-6K predicted by the Pep-fold3. The helical, coil, and extended conformation are coloured by red, blue, and green, respectively.

Brevinin-2GUb was extracted from the skin secretion of *Hylarana guentheri,* and the sequence was attained from a previous paper [14]. In addition, several analogues (Table 1) were rationally designed based on the common characteristics of AMPs and the predicted secondary structure of peptides (Figure 1) to explore the structure–activity relationship and enhance its antimicrobial activity.

According to the results of previous studies, the Rana box at the C-terminal end of peptides in the brevinin-2 family has a slight influence on their bioactivities, and the removal of it can even enhance the antimicrobial activity of the peptides [18]. Therefore, the Rana box domain at the C-terminus was removed, and a C-terminal amidation, which was shown to improve antimicrobial activity [19], was introduced into the sequence. This truncated analogue was named t-Brevinin-2GUb (tB2U). The number of net charges and hydrophobicity was lower than Brevinin-2GUb, while the amphipathicity was increased. Short peptides were reported to have better stability and can be easily absorbed into the body, and their cost is much lower than longer ones [20]. It is well known that the conformation is one of the important features [21] for the bioactivities, so that based on the predicted secondary structure (Figure 1), the main α-helical domain was synthesised as a new truncated analogue called t-Brevinin-2GUb-α (tB2U-α). It had no positive charge in its sequence, but the hydrophobicity and hydrophobicity moment were much higher than those of the parent peptide. It is generally known that the cationic charge plays an indispensable role in regulating the bioactivities of peptides [8], so the negatively charged amino acids in the sequence were replaced by lysine (Lys). The new derivative was named tB2U-K, which possessed +4 net charges and higher hydrophobicity and amphipathicity than Brevinin-2GUb. To explore the significance of the N-terminal end in the sequence, two short peptides, 14-t-Brevinin-2GUb-K (14-tB2U-K) and 7-t-Brevinin-2GUb-K (7-tB2U-K), were designed. As tB2U-K was prone to self-assemble, the threonine, which has the function to cause self-assembly [22], was replaced by a cationic amino acid, Lys, and the analogue was called tB2U-6K. The number of positive charges in the sequence was six, which was the highest one among all the peptides and its amphipathicity was higher than most of the peptides, except for 14-tB2U-K.

### 2.2. Peptide Purification and Identification

The Brevinin-2GUb and its analogues were synthesised by solid-phase peptide synthesis (SPPS) and purified by reverse-phase high-performance liquid chromatography (RP-HPLC). The RP-HPLC chromatogram of Brevinin-2GUb and its derivatives are shown in Figure S1. The molecular mass of the purified peptides was identified by matrix-assisted laser desorption/ionisation time-of-flight mass spectrometry (MALDI-TOF MS). The mass spectra of the peptides are shown in Figure S2. The molecular masses of the synthetic peptides were consistent with those calculated. Therefore, it confirmed that the synthetic peptides were the Brevinin-2GUb and its derivatives, as expected.

### 2.3. Secondary Structure Analysis

**Figure 2:**
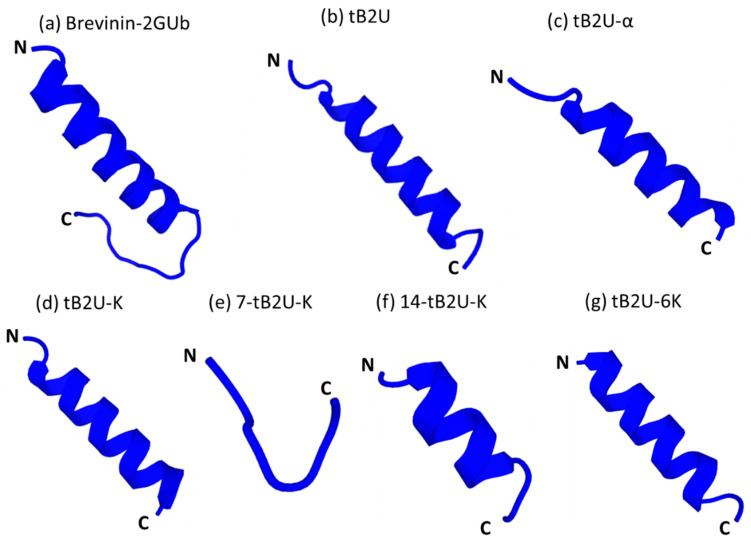
The 3D models of (**a**) Brevinin-2GUb, (**b**) tB2U, (**c**) tB2U-α, (**d**) tB2U-K, (**e**) 14-tB2U-K, (**f**) 7-tB2U-K, and (**g**) tB2U-6K predicted by the Pep-fold3.

**Figure 3:**
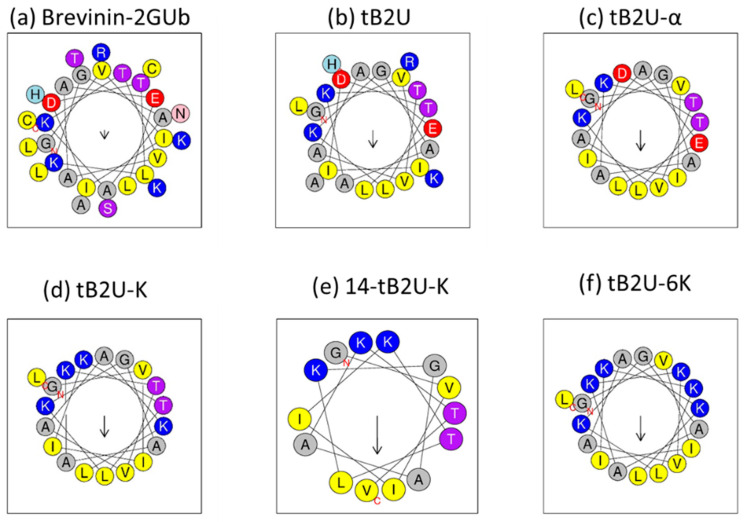
The helical wheel representations of (**a**) Brevinin-2GUb, (**b**) tB2U, (**c**) tB2U-α, (**d**) tB2U-K, (**e**) 14-tB2U-K, and (**f**) tB2U-6K predicted by Heliquest. The hydrophobic, hydrophilic, positively-charged, and negatively-charged amino acids are coloured by yellow, purple, blue, and red, respectively.

The secondary structures of Brevinin-2GUb and its derivatives were predicted by Pep-fold3 (Figure 1) and the 3D models of these peptides are shown in Figure 2. The helical wheel projections were constructed and analysed by Heliquest (Figure 3). The Ramachandran plots of these peptides, which are shown in Figure S3, were made by PROCHECK (the plot of 7-tB2U-K cannot be made due to its short sequence). The exact secondary structures were determined by circular dichroism (CD) (Figure S4) and the helicity percentages were calculated by Bestsel (Figure S5), which are listed in Table 2. In an aqueous environment (20 mM NH_4_Ac), Brevinin-2GUb and its analogues, except for 7-tB2U-K and 14-tB2U-K, can form α-helical conformation with one positive band at 190 nm and two negative bands at 208 and 222 nm, and the percentage of this structure of Brevinin-2GUb was the highest one. On the other hand, all the peptides adopted α-helix in the membrane mimicking environment (50%TFE in NH_4_Ac), and the extent of the α-helical conformation becomes higher than that in the aqueous environment. The change in the helix proportion of tB2U-K was the most obvious one. 

### 2.4. Antimicrobial Assays

The minimum inhibitory concentrations (MICs) and minimum bactericidal concentrations (MBCs) values (Table 3) showed that Brevinin-2GUb has modest activities to inhibit the growth or to kill the Gram-negative bacteria. Moreover, when the concentration reached 512 µM, it could not completely kill the Gram-positive bacteria, except *Staphylococcus aureus (S. aureus*). After the modifications, the tB2U, tB2U-α, 14-tB2U-K, and 7-tB2U-K almost lost their antimicrobial activities against pathogens at concentrations under 512 µM. On the contrary, compared with the parent peptide, tB2U-K and tB2U-6K had a stronger and broad-spectrum antimicrobial activity to kill different pathogens because they also exerted the antimicrobial activity against Gram-positive bacteria; tB2U-6K, in particular, was quite potent. The tB2U-K and tB2U-6K displayed weak activities against *Candida albicans (C. albicans*). In terms of the clinical isolates, Brevinin-2GUb and tB2U-K only possessed the ability to inhibit the growth of *E. coli* (ATCC BAA 2340) and *Klebsiella pneumoniae* (ATCC BAA-1705) (*K. pneumoniae*), while the efficacy of tB2U-K was higher. The tB2U-6K had the effect of killing all the tested clinical strains, and the activity was much stronger than Brevinin-2GUb. The tB2U, tB2U-α, 14-tB2U-K, and 7-tB2U-K nearly had no antimicrobial activity against these strains under the selected concentrations. The therapeutic index (TI) of tB2U-6K was much higher than that of other peptides.

### 2.5. Determination of Cytotoxicity

#### 2.5.1. Haemolysis Assay

**Figure 4:**
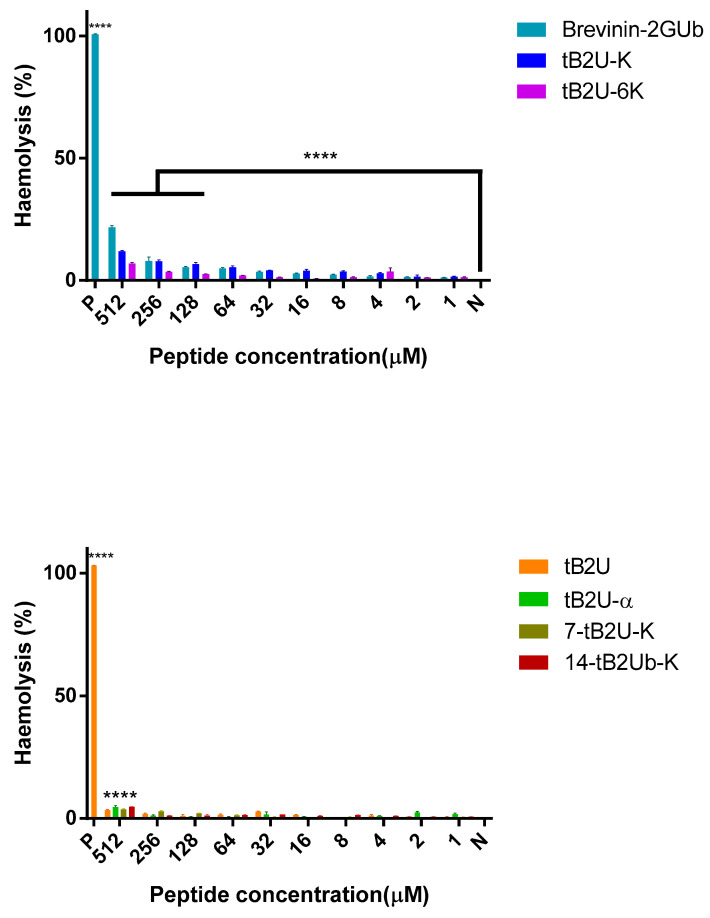
The haemolytic activities of Brevinin-2GUb and its analogues. N represents the negative control and P represents the positive control, which is PBS and 0.1% Triton X-100 in this experiment, respectively. The results were analysed by two-way ANOVA and Dunnett’s multiple comparisons test that compared haemolysis of various concentrations of peptides with negative control. The significance (Table S1) is indicated by ****, which means *p* < 0.0001. The error bar represents the standard error of the mean (SEM) of the nine replicates from three independent tests.

Brevinin-2GUb has relatively weak haemolytic activity against horse blood cells. Compared with the results of Brevinin-2GUb, the haemolytic activities of all the analogues, including tB2U-K and tB2U-6K, which had strong antimicrobial activities and antibiofilm activities, were decreased, and they had only slight haemolysis on horse blood cells, even at the highest tested concentration (Table 3 and Figure 4).

#### 2.5.2. MTT Assay on HaCaT

**Figure 5:**
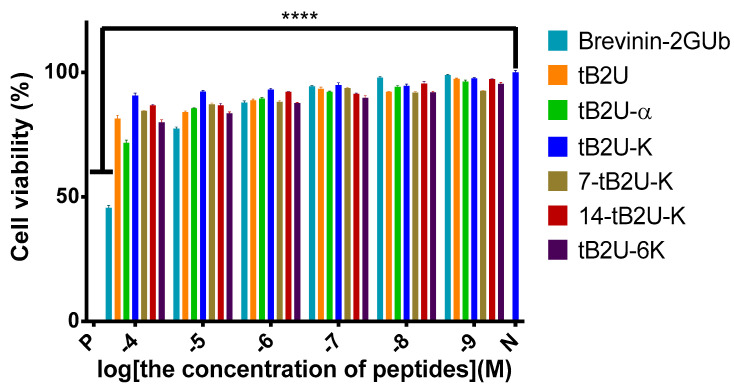
The cytotoxicity of Brevinin-2GUb and its designed derivatives on HaCaT. N represents the negative control and P represents the positive control, which is PBS and 0.1% Triton X-100 in this experiment, respectively. The results were analysed by two-way ANOVA and Dunnett’s multiple comparisons test compared the cell viability of different concentrations of peptides with that of the negative control. The significance (Table S2) is indicated by ****, which means *p* < 0.0001. The error bar represents the SEM of the nine replicates from three independent tests.

MTT assays on HaCaT cells were conducted to determine the cytotoxicity of Brevinin-2GUb and its analogues (Figure 5). The tB2U and tB2U-α only modestly inhibited the growth of these cells even at the highest tested concentration. The other four peptides had little influence on the HaCaT cells. However, the IC_50_ (Table 4) of the original peptide, Brevinin-2GUb, against HaCaT was 68 µM, which demonstrated that it had the most potent cytotoxic effect on the normal cells at the highest tested concentration among these peptides.

### 2.6. Anti-Biofilm Assay

To further explore the biological activities of these peptides, antibiofilm activities against a series of bacteria were investigated (Table 5). The Gram-positive bacteria, *S. aureus,* MRSA and *E. faecalis*, and Gram-negative bacteria, *E. coli, K. pneumoniae,* and *P. aeruginosa*, were selected for antimicrobial assay. Brevinin-2GUb could only inhibit the formation of *S. aureus*, MRSA and *E. coli* biofilms at a relatively high concentration, and it nearly had no biofilm eradication activity at concentrations less than 512 µM. The tB2U, tB2U-α, 14-tB2U-K, and 7-tB2U-K had no antibiofilm activity even at the highest test concentration. The minimum biofilm inhibitory concentration (MBIC) values of tB2U-K and tB2U-6K were much lower than those of Brevinin-2GUb, which showed that they had enhanced activity to inhibit the formation of biofilms, while they nearly had no abilities to eradicate the formed biofilm, even at the highest test concentration.

### 2.7. Time-Killing Assay

**Figure 6:**
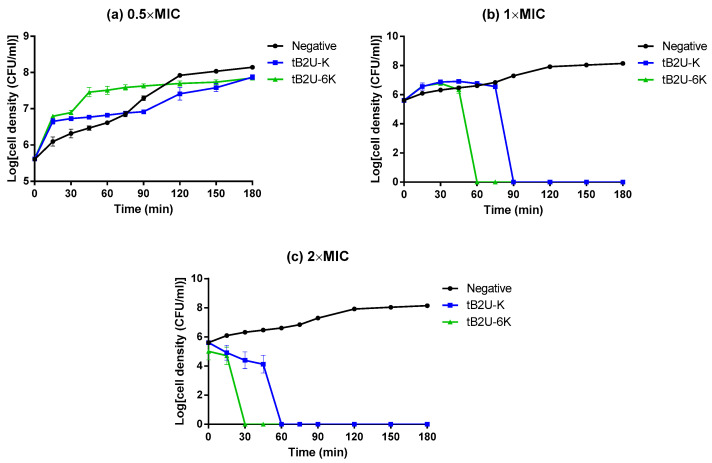
The kinetic time-killing curves of tB2U-K (blue) and tB2U-6K (green) at the concentrations of (**a**) 0.5 × MIC (16 µM), (**b**) 1 × MIC (32 µM) and (**c**) 2 × MIC (64 µM). The bacteria treated with MHB medium only is used as the negative control. The error bar represents the SEM of the three replicates from three independent tests.

As the MIC and MBC of tB2U-K and tB2U-6K against *S. aureus* were entirely the same, to compare the antimicrobial efficiency of these two peptides, the time-killing assay against *S. aureus* was carried out. The peptide concentrations of 0.5 × MIC (16 µM), 1 × MIC (32 µM) and 2 × MIC (64 µM) were adopted. The time-killing curves (Figure 6) demonstrated that at the concentration of 0.5 × MIC, tB2U-K and tB2U-6K could not inhibit the growth of *S. aureus*. However, both peptides could kill the bacteria at 1 × MIC and 2 × MIC within 100 min, which was consistent with the MBC results. At the condition with the same concentration, tB2U-6K can kill *S. aureus* within a shorter time than tB2U-K.

### 2.8. Membrane Permeability Kinetic Assay

**Figure 7:**
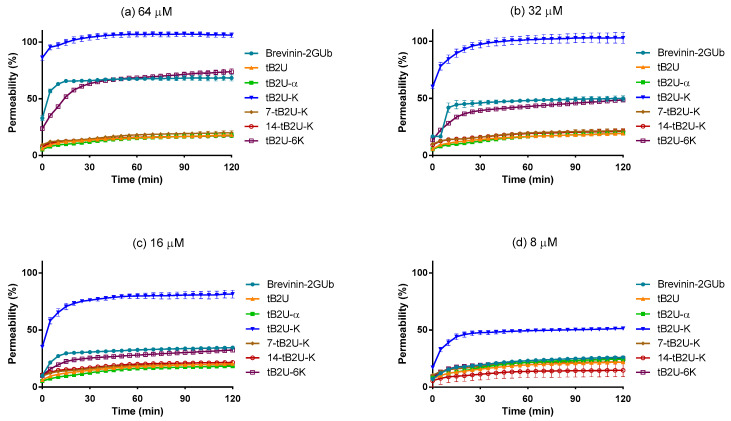
The membrane permeability curves against *S. aureus* of Brevinin-2GUb and its analogues at the concentration of (**a**) 64 µM, (**b**) 32 µM, (**c**) 16 µM and (**d**) 8 µM. The error bar represents the SEM of the five replicates.

The kinetic membrane permeability assay against *S. aureus* was conducted to explore the mechanism of antimicrobial activity (Figure 7). Based on the MICs against *S. aureus* of Brevinin-2GUb, tB2U-K and tB2U-6K, 64 µM, 32 µM, 16 µM, and 8 µM were selected as the concentrations in the experiment. According to the curves, tB2U, tB2U-α, 7-tB2U-K, and 14-tB2U-K had no capacity to permeate the membrane of *S. aureus*. However, at the concentrations of MIC and 2 × MIC, Brevinin-2GUb, tB2U-K and tB2U-6K could cause membrane permeability, and the permeability of tB2U-K was 100%, which was much higher than that of the other two peptides. However, tB2U-K can lead to 80% and 50% membrane permeability at the concentrations of 16 and 8 µM, respectively, while Brevinin-2GUb and tB2U-6K were unable to permeate the membrane at the same concentrations.

### 2.9. Evaluation of Efficacy against E. coli In Vivo

**Figure 8:**
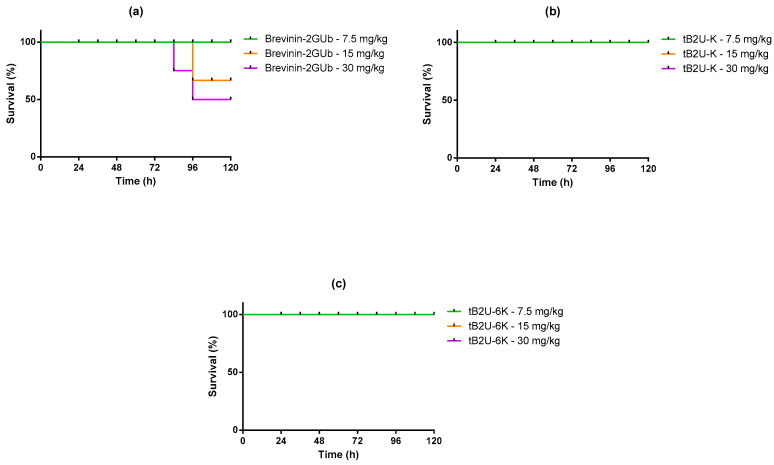
The curves of the survival rate of the waxworms treated with (**a**) Brevinin-2GUb, (**b**) tB2U-K and (**c**) tB2U-6K, separately.

**Figure 9:**
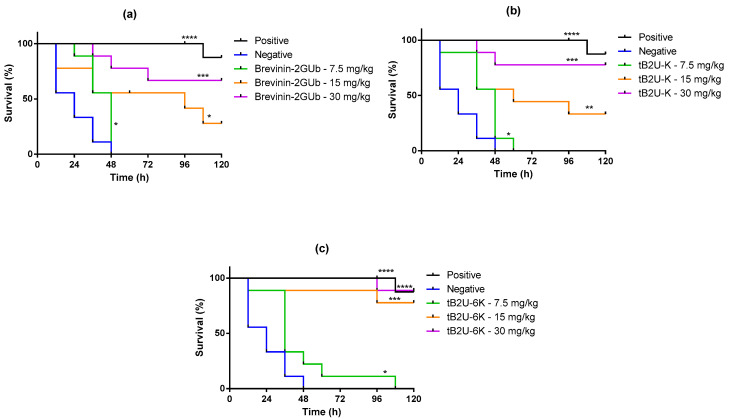
The curves of survival of (**a**) Brevinin-2GUb, (**b**) tB2U-K, and (**c**) tB2U-6K in wax moth infected by *E. coli*. The PBS and 20 mg/kg norfloxacin are selected as the negative and positive controls, respectively. The data were analysed by the survival analysis and curve comparison test comparing the survival rate of different concentrations of peptides with that of the negative control group. The significance (Table S3) is indicated by the **** (*p* < 0.0001), *** (*p* < 0.001), ** (*p* < 0.01), and * (*p* < 0.05).

According to the relatively low MBCs of tB2U-K and tB2U-6K against *E. coli*, the wax moth (*Galleria mellonella*) infected by *E. coli* was selected as the model to verify the antimicrobial activity of these peptides in vivo. The 7.5, 15, and 30 mg/kg were chosen as the test concentrations based on the MBC values. The toxicities of Brevinin-2GUb, tB2U-K, and tB2U-6K with the above concentrations were examined at the first step, and the survival curves (Figure 8) indicated that the two analogues were harmless to the waxworms due to the 100% survival rate while Brevinin-2GUb had modest toxicity to *E. coli* when the concentration reached 15 mg/kg. The mortality of worms infected by *E. coli* was dramatically decreased after treating the peptides compared with PBS (Figure 9). The higher the concentrations of the peptides, the more wax moths could be saved. At the lowest test concentration, these three peptides displayed a similar ability to combat the bacteria, while at 15 and 30 mg/kg, tB2U-6K exerted higher efficacy against *E. coli* than Brevinin-2GUb and tB2U-K. Compared with the parent peptide, tB2U-K attained a slight improvement in the activity to combat *E. coli*. The antimicrobial activity of tB2U-6K at the highest test concentration, the strongest among the three peptides, was similar to 20 mg/kg norfloxacin.

## 3. Discussion

At present, antibiotic resistance, which causes treatment failures, has become one of the major threats to the development of modern medicine; AMPs are compounds that have the potential to deal with this worldwide problem due to their advantages [25]. The peptides in the brevinin-2 family have been widely studied, and they were strong candidates to the clinical antimicrobial agents, owing to their broad-spectrum bioactivity and low toxic effects [26]. Furthermore, Brevinin-2GUb, which was extracted from the skin secretion of *Hylarana guentheri*, was reported to possess antimicrobial activity against several microbes [14], while there has been no more in-depth research on this. Therefore, Brevinin-2GUb was selected as the subject of this study.

The conformation of AMPs is known as one of the significant features for their antimicrobial activities and they have been categorised into several groups according to their secondary structure, and most of the AMPs adopt α-helix and β-sheet [21]. The structure of magainin-2 in an aqueous environment is random coil, while, when it is attached to the bacterial membrane, magainin-2 transforms its conformation into an α-helix [27]. From the CD spectra, Brevinin-2GUb belongs to the α-helical group while different from other peptides, Brevinin-2GUb adopts the α-helical conformation in both aqueous and membrane-mimicking environments. The percentage of α-helix of Brevinin-2GUb and its analogues in the 50% TFE in NH_4_Ac is much higher than in 20 mM NH_4_Ac, revealing that the conformations of these peptides become more solid when they are attached to the bacterial membrane. Moreover, common features, including sequence length, net charges, and hydrophobicity, are not separated from each other [9], so that they influence the formation of secondary structure. Comparing the secondary structure of Brevinin-2GUb, tB2U, and tB2U-α, with the truncation on the parent peptide, the extents of the α-helix of the analogues become lower and lower due to the shorter length, which makes the peptide difficult to form their secondary structure. However, when the cationic charges are introduced into the sequence, tB2U-K and tB2U-6K have higher amphipathicity and a higher possibility to form an α-helical conformation than tB2U-α with the same number of amino acids. Nevertheless, tB2U-6K, which contains +6 net charges, is less likely to form α-helix than tB2U-K, which possesses +4 net charges. The damage on the α-helix can be explained by the steric hindrance and the repulsion of cationic charges caused by the consecutive Lysine [28]. Therefore, the increase of positive charges has little effect on its secondary structure when it reaches a certain range. Except for the relatively short sequence length, the disruption of the hydrophobic face is the main reason for the low tendency of 7-tB2U-K and 14-tB2U-K to transform into α-helical conformation.

The Rana box is a loop motif, which is highly conserved in many peptide families, such as brevinin-1, brevinin-2, nigrocin, ranatuerin-2, esculentin-1, etc., and it consists of two cysteines, which can form a disulphide bridge, with the second one locating at the C-terminal end of the sequence. In the middle of these two cysteines, there are usually four or five residues [12]. However, the function of this motif is diverse in different peptide families: the removal of the Rana box can reduce the bioactivity of the peptide dramatically in ranatuerin-2 and brevinin-1 [29], while the presence of this motif has little influence on the peptides in esculentin-1 [30]. Chen et al. [18] discovered that the lack of the Rana box in brevinin-2GHk could even improve its antimicrobial activity. Based on the results of the antimicrobial assay, although the removal of the Rana box leads to the loss of antimicrobial activity of tB2U, the capacity to combat pathogens becomes stronger when the cationic charges and C-terminal amidation are added into the sequence (tB2U-K), which illustrated that Rana box is not indispensable for the antimicrobial activity of Brevinin-2GUb. The reason for the weaker antimicrobial activity of tB2U and tB2U-α may be the loss of the net charges and the low tendency of forming α-helical conformation. Stigmurin, a peptide from the scorpion, has weak activity to kill *S. aureus*, *Staphylococcus epidermidis,* and *C. albicans,* while its derivatives, whose amino acids were replaced with lysine, have the capacity to influence the growth of Gram-negative bacteria and fungi [31]. Previous studies concluded the amphipathicity is a critical factor for the peptides with antibacterial and antifungal activity by the large-scale analysis on the large available AMP peptidome [10]. Comparing the MICs and MBCs of these peptides, the increase of net charges can considerately improve the activity and efficiency to kill bacteria and can generate the effect to combat multidrug-resistant bacteria, clinical isolates, and fungi. Brevinin-2GUb has no influence on the growth of MRSA, *E. faecalis,* and *C. albicans,* while the activity of tB2U-K and tB2U-6K to kill these pathogens emerged with the introduction of lysine, the increase of the amphipathicity, which means the component possesses both hydrophilic and hydrophobic residues [32], and the high potential of them to adopt α-helix in the membrane-mimic environment. Moreover, the antimicrobial activity of tB2U-6K against clinical strains is more potent than other analogues, which reveals that when the number of net charges reaches a certain range, the peptide can have a stronger ability to fight clinical isolates. This improvement makes tB2U-6K more possible to deal with the problems resulting from antibiotic resistance. The capacity of tB2U-6K is just slightly improved over that of tB2U-K. This result is because the hydrophobicity of the peptide is not high enough to deepen the insertion into the bacterial membrane when the number of net charges reaches a threshold so that the effect cannot be improved as significantly as before. Meanwhile, the replacement of the threonine makes the tB2U-6K less likely to self-assemble into nanostructures via removing the formation of the disulphide bond. The study on the LBU illustrated that maintaining the hydrophobic face is a significant factor in the ability against microbes [33]. The antimicrobial activity of the 7-tB2U-K and 14-tB2U-K is lost with the truncated modification, which is caused by the damage of the hydrophobic face and the pretty short sequence that cannot form an α-helical conformation (Figure 2), which has the function of enhancing the ability to interact with the bacterial membrane. Thus, the 1st to the 19th amino acids at the N-terminal end, which contains the entire hydrophobic face, is the core structure of Brevinin-2GUb on its antimicrobial activity.

Previous research concluded that AMPs exert their antimicrobial activities through diverse mechanisms. Temporin B and temporin L exert their antimicrobial activities by perturbing the bacterial membrane via a barrel-stave model [34]. Some peptides interact with the intracellular targets to inhibit key processes, such as proline-rich AMP, pyrrhocoricin fights the bacteria by inhibiting protein synthesis, and buforin 2 can translocate across the bacterial membrane without disrupting it and bind with the intracellular DNA or RNA [35]. Brevinin-2GUb, tB2U-K, and tB2U-6K are considered to be attracted by the bacteria through the positive charges and interact with them to permeabilise their membranes, leading to death. However, the exact mode of the mechanism is still unknown.

Brevinin-2GUb has a relatively low haemolysis activity and has modest cytotoxicity against the normal cell line, HaCaT. As most of the peptides kill the bacteria by disrupting the membrane, they have the possibility of interacting with the mammalian cell membrane as well, which will bring cytotoxicity [36]. Therefore, the higher hydrophobicity and amphipathicity will result in a more powerful haemolysis activity [36]. A previous study showed that brevinin-2PRc and brevinin-2PRd attained low toxicity against red blood cells due to the reduced hydrophobicity with the substitution of Phe with Leu [37]. Nevertheless, although the hydrophobicity of the analogues is higher than that of Brevinin-2GUb, apart from tB2U-6K, the haemolysis activity is much lower than that of the parent peptide. Except for the peptides without potent antimicrobial activity, tB2U-K and tB2U-6K contain powerful selectivity and this is generated by the increase of the cationic charges, which makes the electrostatic interaction between the peptides and bacterial membrane stronger, so that the peptide has more powerful affinity with the negatively-charged bacterial membrane [8], and the deletion of the Rana box, like nigrocin-HLM, which eliminates the Rana box from the nigrocin-HL, achieves a significantly improved bioactivity and a much weaker toxic effect [12]. The toxicity is a key factor to evaluate the cell selectivity of the peptide and can be used to calculate the therapeutic index (TI), which is a quantitative measurement for the relative safety of the peptide [38]. The higher TI value represents a higher specificity to the microbes, which means it is safer [9]. For example, the TI of diazepam, which is viewed as a relatively safe drug, is 100:1 [39], while some antibiotics only have a narrow therapeutic window, such as gentamicin and vancomycin [40]. However, the TI of tB2U-6K is 158.02, which is the highest among these peptides. The relatively high TI value reveals the security of tB2U-6K is similar to the clinical drugs, which gives the peptide more potential to be developed into clinical use.

Due to the influence of physiological factors, there may be some difference between the antimicrobial activity in vitro and in vivo [41]. Consistent with the haemolysis assay and cytotoxicity assay results, Brevinin-2GUb is much harmful to the wax moth than tB2U-K and tB2U-6K. A previous study demonstrated that the Indolicidin could significantly improve the survival rate, up to 95%, of *Galleria mellonella* infected by the enteroaggregative *E. coli* [42]. In our study, with the increase of the number of net charges, the survival rate of *E. coli*-infected wax moth is enhanced and the ability of tB2U-6K with the highest test concentration in vivo, whose mortality rate is just 11.11% within five days, is similar to the effect of norfloxacin with the concentration of 20 mg/kg, which makes tB2U-6K more potential to exert equal activity against pathogens in the physiological environment.

Overall, the increase of net charges can enhance the activity of peptides against antibiotic-resistant bacteria and clinical isolates, and the improved amphipathicity can enhance the propensity of peptides to contain both antibacterial and antifungal activity. Therefore, it is critical to explore the balance between activity and toxicity, which depends on the ratio between net charges and hydrophobicity when optimising the peptides. In addition, shortening the peptides as much as possible, while maintaining their active fragments is a practical strategy for modifying the AMPs. It can reduce the cost of the chemical synthesis of peptides as well.

In general, the tB2U-6K contains the most potent antimicrobial activity against different pathogens, especially antibiotic-resistant clinical bacteria, through membrane permeabilisation and higher efficiency, to kill microbes among these peptides. Meanwhile, its powerful selectivity and wide therapeutic window give it more potential to be a candidate for development of novel antimicrobial agents.

## 4. Materials and Methods

### 4.1. Peptide Synthesis

The sequence of Brevinin-2GUb was attained from a previous article [14]. The peptides, Brevinin-2GUb and its analogues, were synthesised by SPPS through use of a Tribute^®^ 2 channel peptide synthesiser (Protein Technologies, Tucson, AZ, USA). First, the amino acids of each sequence were weighed and put in the vial together with an activator, hexafluorophosphate benzotriazole tetramethyl uronium (HBTU). Next, the rink amide resin, which was weighed accurately, was deprotected by 20% piperidine in dimethylformamide (DMF) and coupled with the amino acid, activated through N-methylmorpholine (NMM) and HBTU, from the C-terminus to N-terminus. Then, the resin and protection groups in the side chains were cleavaged by cleavage solution, which contains 94% trifluoroacetic acid (TFA), 2% water, 2% thioanisole (TIS), and 2% 1,2-ethanedithiol (EDT) and washed by diethyl ether three times. Finally, the peptides were lyophilised in a freeze dryer for 48–50h, and the dry peptides were stored at −20 ℃.

### 4.2. Peptide Purification

The lyophilised peptides were dissolved in buffer A (99.95% ddH_2_O and 0.05% TFA) to make a concentration of 5 mg/mL. After centrifugation for clarification, 1 mL of the solution was injected into the RP-HPLC, which contained a Jupiter C18 column (Phenomenex, Macclesfield, Cheshire, UK) of the size 250 × 21.2 mm. Then, the crude peptides were eluted by gradient elution with buffer A (99.95% ddH_2_O and 0.05% TFA) and buffer B (80% acetonitrile, 19.95% ddH_2_O and 0.05% TFA) based on the polarity discrepancy of each fragment. The flow rate was 8 mL/min, and the wavelength was set at 214 nm. The whole length of the elution process was 60 min.

### 4.3. Peptide Identification

The peptide fragments were analysed using MALDI-TOF MS on a linear time-of-flight Voyager DE mass spectrometer in positive detection mode using α-cyano-4-hydroxycinnamic acid (CHCA) as the matrix. Two µg of each fragment were loaded and spotted onto the MALDI ground-steel target plate and then air-dried. Afterwards, 1 µL excess matrix solution (10 mg/mL) was loaded to the plate and then air-dried, as well. The peptides were identified by the appropriate mass measurement, which depended on the mass-to-charge (*m/z*) ratio.

### 4.4. Secondary Structure Prediction and Determination

The secondary structures of the peptides were predicted by Pep-fold3 (https://bioserv.rpbs.univ-paris-diderot.fr/services/PEP-FOLD3/, accessed on 16 July 2021), and their physicochemical properties were calculated by Heliquest (https://heliquest.ipmc.cnrs.fr/, accessed on 26 June 2021). The validation of these structures was presented by the Ramachandran plots of these peptides, which were made by PROCHECK (https://www.ebi.ac.uk/thornton-srv/software/PROCHECK/, accessed on 16 July 2021).

The exact secondary structure was determined by CD using a JASCO J-815 CD spectrometer (Jasco, Essex, UK). The samples were the peptides dissolved in different environmental solutions, including NH_4_Ac and 50% TFE in NH_4_Ac, with a concentration of 100 µM. The temperature was 20 ℃ and the scanning speed was 100 nm/min with 1 nm bandwidth and 0.5 nm data pitch. The wavelength of the CD analysis was from 190 to 250 nm. The helicity percentage of these peptides were analysed by the online tool, BeStSel (https://bestsel.elte.hu/index.php, accessed on 16 July 2021).

### 4.5. Antimicrobial Assay

The method of antimicrobial assay is based on the Methods for Dilution Antimicrobial Susceptibility Tests for Bacteria that Grow Aerobically from the Clinical and Laboratory Standards Institute (CLSI).The antimicrobial activities of peptides were evaluated against Gram-positive bacteria *S. aureus* (ATCC 6538), MRSA (NCTC 12493), *E. faecalis* (NCTC 12493), Gram-negative bacteria *E. coli* (ATCC CRM 8739), *K. pneumoniae* (ATCC 43816), *P. aeruginosa* (ATCC CRM 9027), yeast *C. albicans* (ATCC CRM 10231) and clinical isolates MRSA (B042 V2E1 A), *E. coli* (ATCC BAA-2340), *K. pneumoniae* (ATCC BAA-1705), *P. aeruginosa* (B004 V2S2 B).

At the first stage of the assay, 20 μL bacteria were cultured in 100 mL Mueller Hinton Broth (MHB) or Yeast Extract–Peptone–Dextrose broth (YPD-B), respectively, and was incubated overnight. Then, microorganisms were subcultured in 20 mL fresh MHB or YPD-B to achieve cultures in the logarithmic phase (1 × 10^8^ CFU/mL), which can be verified by optical density (OD) (The OD of Gram-positive bacteria is 0.23, OD of Gram-negative bacteria is 0.4, and OD of yeast is 0.15), and viable cell counts. Then, the medium was used to dilute the cultures to specific cell densities (Gram-negative and Gram-positive bacteria: 5 × 10^5^ CFU/mL; yeast: 1 × 10^6^ CFU/mL), which were the final concentrations of bacterial suspensions used in the assay. The peptides were dissolved in DMSO to reach a final concentration of 512 µM. A series of two-fold dilutions ranging from 512 µM to 1 µM were dosed in the 96-well plates. 

Antimicrobial assays were conducted in 96-well plates, which were divided into five groups, including a negative control group (PBS), a positive control group (20 μg/mL norfloxacin for bacteria and 10 μg/mL amphotericin B for yeast), vehicle control (1% DMSO), blank control (MHB or YPD-B), and a peptide group. Each microorganism had five repetitions, and each well contained 1 µL corresponding solution and 99 µL culture. After incubation at 37 °C overnight, the absorbance of each well was tested by a microplate reader (EL808, Biolise BioTek Winooski, Minneapolis, USA) under a 550 nm wavelength. The MIC is the lowest concentration at which no bacterial growth can be observed.

The equation to calculate the cell viability is shown below.

(1) $$Cell\;viability\;\left(\%\right)=\left(A-A_{b}\right)/\left(A_{n}-A_{b}\right)\times 100\%$$

A represents the absorbance of each well in the sample groups. $A_{b}$ represents the average absorbance of the wells in the blank control group. $A_{n}$ represents the average absorbance of the wells in the negative control group.

The next day, 10 µL of every clear well was taken out to be cultured on a Mueller Hinton Agar (MHA) plate and incubated at 37 °C for 16–20 h. The MBC is the lowest concentration without any bacterial colonies, which means at this concentration, the peptide reduces the viability of the pathogen by more than 99.9%.

### 4.6. Determination of Cytotoxicity

#### 4.6.1. Haemolysis Assay

The haemolysis assay was conducted on defibrinated horse blood (TCS Biosciences Ltd., Buckingham, UK), which was washed by PBS in advance. The peptides were dissolved in PBS to the concentration of 1024 μM, and a series of two-fold dilutions was made by PBS as well. A volume of different concentrations of 120 μL peptide solutions were mixed with the same volume of 2% (*v/v*) of horse red cell suspension gently in a 1.5 mL tube, and the complex was incubated at 37 ℃ for 2 h. PBS and 0.1% Triton X-100 with the same volume were used as the negative and positive controls, respectively. Each group had three replicates. After the incubation, 100 μL supernatant of each tube was aspirated into a new 96-well plate, and their absorbances were analysed by a microplate reader (EL808, Biolise BioTek Winooski, Minneapolis, USA) at a wavelength of 570 nm. The equation to calculate the haemolysis of the peptides is shown below.

(2) $$Cell\;viability\;\left(\%\right)=\left(A-A_{n}\right)/\left(A_{p}-A_{n}\right)\times 100\%$$

A represents the absorbance of each well in the sample groups. $A_{n}$ represents the average absorbance of the wells in the negative control group. $A_{p}$ represents the average absorbance of the wells in the positive control group.

#### 4.6.2. MTT Assay on HaCaT

The cytotoxicity of the peptides against the human epidermal keratinocyte line, HaCaT, were tested by MTT cell viability assay. HaCaT was cultured in DMEM medium with 10% Fetal Bovine Serum (FBS; Gibco, UK) and 1% penicillin-streptomycin (10,000 Unit/mL, 10,000 μg/mL; Gibco, Grand Island, NY, USA) and incubated at 37% and 5% CO_2_. A volume of 100 μL cell suspension was seeded in each well of a 96-well plate with the density of 10,000 cells in each well and the plate was incubated at 37 ℃ and 5% CO_2_ overnight. To make the cells in the same stage of mitosis, the medium of cells was transformed into the serum-free medium, and they were incubated for at least 4 h. The peptides were dissolved in ddH_2_O to make a stock solution with a concentration of 10^−2^ mol/L, and a series of 10-fold dilutions from stock solution, whose concentrations were from 10^−4^ to 10^−9^ M, was made by the serum-free medium. After discarding the serum-free medium in the plate, 100 μL of each peptide solution was added into the wells and incubated for 24 h. The 0.1% Triton X-100, 1% ddH_2_O, and serum-free medium were added as positive, vehicle and growth control, respectively. MTT solution (3-(4,5-dimethylthiazol-2-yl)-2,5-diphenyltetrazolium bromide) with a volume of 10 μL was added into each well and the plate was incubated for two hours. After that, the medium was replaced by 100 μL DMSO, and they were put in the shaking incubator for 15 min. The absorbance of each well was analysed by a microplate reader (EL808, Biolise BioTek Winooski, Minneapolis, USA) at a wavelength of 570 nm. The equation to calculate the cell viability of cancer cells is shown below.

(3) $$Cell\;viability\;\left(\%\right)=\left(A-A_{b}\right)/\left(A_{n}-A_{b}\right)\times 100\%$$

A represents the absorbance of each well in the sample groups. $A_{b}$ represents the average absorbance of the wells in the blank control group. $A_{n}$ represents the average absorbance of the wells in the negative control group.

### 4.7. Anti-Biofilm Assay 

The anti-biofilm activities of the peptides against *S. aureus* (ATCC 6538), MRSA (NCTC 12493), *E. faecalis* (NCTC 12493), *E. coli* (ATCC CRM 8739), *K. pneumoniae* (ATCC 43816), and *P. aeruginosa* (ATCC CRM 9027) were evaluated.

#### 4.7.1. MBIC

The bacteria were inoculated in a different medium (Gram-positive bacteria were in TSB and Gram-negative bacteria were in LB) overnight, and the subculture was conducted in the same medium. When the bacteria reached a specific density, 100 µL of the dilution was added to each well of the 96-well microplate. Then 1 µL of peptide at a series of concentrations, the same concentration in the antimicrobial assay, were added into the well together with the bacteria. The plates were put into a moist shaking incubator with a speed of 200 rpm, and the temperature was set at 37 ℃. After a 24-h incubation, the liquid in the wells was aspirated, and each was washed twice by 100 µL PBS. One hundred µL of methanol were added into each well to stabilise the biofilm. After air-drying, 100 µL crystal violet was used to dye the biofilm, and they were dissolved in the 100 µL glacial acidic acid after drying. The solutions were transferred to a new microplate, and the absorbance of each well was tested by a microplate reader (EL808, Biolise BioTek Winooski, Minneapolis, MN, USA) at 595 nm wavelength. MBIC was the minimum concentration of the peptide showing no biofilm formation.

#### 4.7.2. MBEC

The bacteria were inoculated in a different medium (Gram-positive bacteria were in TSB and Gram-negative bacteria were in LB) overnight, and the subculture was conducted in the same medium. When the bacteria reached a specific density, 100 µL of the dilution was added to each well of the 96-well microplates. The plates were put into a moist shaking incubator with a speed of 200 rpm and the temperature was 37 ℃. After a 24 h incubation, the liquid in the wells was aspirated and they were washed twice by 100 µL PBS of each well. The peptides with the same concentrations were diluted in the corresponding medium and 100 µL were added into each well. After a 24-h incubation, the liquid in the wells was aspirated and they were washed twice by 100 µL PBS. One hundred µL of methanol were added into the wells to stabilise the biofilms. After being air-dried, 100 µL crystal violet was used to dye the biofilm, and they were dissolved in the 100 µL glacial acidic acid after drying. The solutions were transferred to a new microplate, and the absorbance of each well was tested by a microplate reader (EL808, Biolise BioTek Winooski, Minneapolis, USA) at 595 nm wavelength.

### 4.8. Time-Killing Assay

*S. aureus* (ATCC 6538) was inoculated in the MHB medium at 37 ℃ overnight. Then 500 µL of culture was used to subculture in the MHB medium to reach a logarithmic growth phase (OD = 0.23 at 550 nm). Then, it was diluted by MHB medium to obtain a specific cell density (5 × 10^5^ CFU/mL). The peptides at concentrations of 0.5 × MIC, 1 × MIC and 2 × MIC were mixed with the bacterial cultures for 3 h. Aliquots were aspirated from the tube at 0, 15, 30, 45, 60, 75, 90,120, 150, and 180 min. The removed contents were diluted by PBS and seeded onto the MHA plate. The colonies were counted after overnight incubation at 37 ℃.

### 4.9. Membrane Permeability Kinetic Assay

The membrane permeability activity was evaluated by the SYTOX™ Green nucleic acid stain (Life Technologies, Carlsbad, CA, USA). *S. aureus* was inoculated in TSB overnight, and the subculture was conducted in the same medium for 2.5 h to reach a logarithmic growth phase. Then, the culture was centrifuged to collect the cells at the bottom of the tube, and 30 mL of 5% TSB in 0.85% NaCl were used to wash the bacterial cells twice. The concentrations of 8, 16, 32, and 64 µM were selected as the working concentrations. Then 40 µL peptide solutions were mixed with 50 µL bacterial cultures and 10 µL of SYTOX Green. Then 8 µM melittin and 5% TSB were utilised as the positive and negative controls, respectively. The fluorescent intensity of each well was tested at 37 ℃ with the excitation at 485 nm and emission at 528 nm by a microplate reader (EL808, Biolise BioTek Winooski, Minneapolis, USA) for 2 h and the interval was 5 min.

### 4.10. Evaluation of Efficacy against E. coli In Vivo

The wax moth (*Galleria mellonella*) model was reported as an ideal model for the screening of antimicrobial activity due to the similar microbial virulence to that in mammalian model, the similar immune response to that of mammalians and the low cost for the high-throughput screening [43,44,45,46,47]. Therefore, the wax moth model was selected for the model of the assay in vivo. The wax moth (Livefood UK Ltd., Rooks Bridge, UK), whose weights were 250 ± 25 mg, were divided into different groups with nine in each group. The 10 µL of suspension of *E. coli*, whose concentration was 5 × 10^6^ CFU/mL, was injected into each worm. After 2 h, 10 µL of the peptide solution at the concentrations of 7.5, 15, and 30 mg/kg, were injected into the worms in the corresponding groups. Then 20 mg/kg norfloxacin and PBS were utilised as the positive and negative controls, respectively. The survival rates of the worms were recorded every 12 h, lasting for 120 h.

### 4.11. Statistical Analysis

All the data from the assays on bioactivity evaluation were analysed using Prism (version 6.0; GraphPad Software Inc., San Diego, CA, USA). The results from the assays in vitro were analysed by two-way ANOVA and Dunnett’s multiple comparisons test. The results from the assay in vivo were analysed by the survival analysis and curve comparison test. The significance was indicated by **** (*p* < 0.0001), *** (*p* < 0.001), ** (*p* < 0.01), and * (*p* < 0.05).

## 5. Conclusions

In conclusion, the N-terminal part of Brevinin-2GUb is critical for its antimicrobial activity, and the 1st and 19th amino acids are the active fragments. The Rana box is not a crucial motif for the bioactivity of Brevinin-2GUb, while the entire hydrophobic face and net charges are the truly significant features. The increase of the cationic charges drastically enhances its bioactivities and weakens the toxicity, so that it is important to discover the optimised ratio between the hydrophobicity and net charges. The higher amphipathicity can broaden the spectrum of the pathogens that the peptides can fight, and the α-helical conformation has a significant influence on the interaction between the peptides and bacteria. The mechanism of the antimicrobial activity is considered membrane permeabilisation. Moreover, shortening the length of the peptide while keeping its bioactivities is a reasonable method for modifying peptides due to the lower cost of the synthesis. Although the antimicrobial activity of tB2U-6K was verified in the wax moth model, it still needs further study on the mammalian models for its clinical use.

## Figures and Tables

**Table 1:** The physicochemical properties of Brevinin-2GUb and its analogues.

Peptide	Sequence	Hydrophobicity <H>	Hydrophobic Moment <μH>	Net Charge (z)
Brevinin-2GUb	GVIIDTLKGAAKTVAAELLRKAHCKLTNSC	0.379	0.144	+3
tB2U	GVIIDTLKGAAKTVAAELLRKAH-NH_2_	0.346	0.288	+2
tB2U-α	GVIIDTLKGAAKTVAAELL-NH_2_	0.501	0.339	0
tB2U-K	GVIIKTLKGAAKTVAAKLL-NH_2_	0.471	0.347	+4
7-tB2U-K ^1^	GVIIKTL-NH_2_	-	-	+1
14-tB2U-K	GVIIKTLKGAAKTV-NH_2_	0.422	0.506	+3
tB2U-6K	GVIIKKLKGAAKKVAAKLL-NH_2_	0.339	0.410	+6

^1^ The hydrophobicity and hydrophobic moment of 7-tB2U-K cannot be analysed by Heliquest due to the short length of the sequence.

**Table 2:** Secondary structure analysis of Brevinin-2GUb and its analogues.

Peptide	20 mM NH_4_Ac			50% TFE in NH_4_Ac		
Name	Helix (%)	Antiparallel (%)	Turn (%)	Helix (%)	Antiparallel (%)	Turn (%)
Brevinin-2GUb	62.9	0.0	6.5	73.2	0.0	4.9
tB2U	58.7	0.0	6.4	72.7	0.0	4.6
tB2U-α	39.9	10.3	12.1	50.3	2.6	10.6
tB2U-K	50.3	0.0	1.1	89.6	0.0	0.0
7-tB2U-K	25.9	23.0	0.0	51.0	8.2	0.0
14-tB2U-K	15.9	23.8	10.9	31.0	18.8	9.1
tB2U-6K	51.5	0.0	7.8	66.0	0.0	3.2

**Table 3:** The MICs and MBCs of Brevinin-2GUb and its analogues ^1^.

Bacteria	MIC/MBC(μM)						
	Brevinin-2GUb	tB2U	tB2U-α	tB2U-K	7-tB2U-K	14-tB2U-K	tB2U-6K
*S. aureus*	32/32	256/>512	>512	32/32	512/>512	256/>512	32/32
*E. coli*	16/16	128/128	>512	8/8	>512	>512	2/2
*C. albicans*	>512	>512	>512	64/64	>512	>512	256/256
*Methicillin-resistant Staphylococcus aureus* (MRSA)	>512	>512	>512	128/128	256/>512	>512	64/128
*K. pneumoniae*	128/128	512/>512	>512	128/128	>512	>512	32/32
*Pseudomonas aeruginosa* (*P. aeruginosa*)	128/512	>512	>512	32/32	>512	>512	16/16
*Enterococcus faecalis* (*E. faecalis*)	>512	>512	>512	32/32	>512	>512	64/64
Clinical strains							
MRSA (B042 V2E1 A)	>512	>512	>512	>512	>512	>512	256/256
*E. coli* (ATCC BAA-2340)	32/32	512	>512	16/16	>512	>512	8/8
*P. aeruginosa* (B004 V2S2 B)	>512	>512	>512	>512	>512	>512	64/64
*K. pneumoniae* (ATCC BAA-1705)	512/512	>512	>512	128/128	>512	>512	128/128
HC_50_ (μM)	2010	16,393	15,609	3056	11,008	10,834	6506
TI ^2,3^ (Overall)	27.98	16.01	15.24	70.18	10.75	10.58	158.02

^1^ The results were achieved from the 15 replicates in the three independent assays. ^2^ Therapeutic index (TI) is the ratio between HC_50_ (µM) and the geometric mean (GM) of MICs (µM), which represents the antimicrobial selectivity of the peptides [23]. ^3^ Because the antimicrobial activity of tB2U, tB2U-α, 7-tB2U-K, and 14-tB2U-K were not detectable even at 512 µM, the value of two times the highest test concentration (1024 µM) was used as the GM of the MICs of these peptides to calculate the TI [24].

**Table 4:** The IC_50_ of Brevinin-2GUb and its analogues against HaCaT cells ^1^.

Normal Cell Lines	IC50 (µM)						
	Brevinin-2GUb	tB2U	tB2U-α	tB2U-K	tB2U-6K	7-tB2U-K	14-tB2U-K
HaCaT	68.0	381.6	228.0	883.2	346.8	485.3	572.6

^1^ The results were achieved from the nine replicates in three independent assays.

**Table 5:** The MBICs and minimum biofilm eradication concentrations (MBECs) of Brevinin-2GUb and its derivatives ^1^.

Bacteria Strains	MBIC/MBEC (μM)						
	Brevinin-2GUb	tB2U	tB2U-α	tB2U-K	tB2U-6K	7-tB2U-K	14-tB2U-K
*S. aureus*	256/>512	>512	>512	16/>512	64/>512	512/>512	>512
*E. coli*	128/>512	>512	>512	16/>512	2/>512	>512	>512
*K. pneumoniae*	>512	>512	>512	128/>512	32/>512	>512	>512
*P. aeruginosa*	>512	>512	>512	128/>512	32/>512	>512	>512
MRSA	256/>512	128/>512	>512	16/>512	32/>512	512/>512	>512
*E. faecalis*	>512	256/>512	>512	32/>512	64/>512	>512	>512

^1^ The results were achieved from the fifteen replicates in three independent tests.

## Data Availability

Not applicable.

## Supplementary Materials

The following are available online at https://www.mdpi.com/article/10.3390/antibiotics10080895/s1, Figure S1. The RP-HPLC chromatograms of (A) Brevinin-2GUb, (B) tB2U, (C) tB2U-α, (D) tB2U-K, (E) tB2U-6K, (F) 7-tB2U-K and (G) 14-tB2U-K. The elution peak of each peptide is indicated by the arrow. Figure S2. The mass spectra of (A) Brevinin-2GUb, (B) tB2U, (C) tB2U-α, (D) tB2U-K, (E) tB2U-6K, (F) 7-tB2U-K and (G) 14-tB2U-K obtained from MALDI-TOF MS. The observed [M + H] ^+^ ion, the sodium and potassium adduct ions are indicated by arrows. Figure S3. The Ramachandran plots of (A) Brevinin-2GUb, (B) tB2U, (C) tB2U-α, (D) tB2U-K, (E) tB2U-6K and (F) 14-tB2U-K predicted by PROCHECK (https://www.ebi.ac.uk/thornton-srv/software/PROCHECK/). The X and Y axis is the torsional angles-Psi (Ψ) and Phi (Φ), respectively. The glycine residues are separately represented by triangles and the red regions are the core areas which represent the most favourable combinations of phi-psi values. Figure S4. The CD spectra of Brevinin-2GUb and its analogues in (A) aqueous solution (20 mM NH4Ac) and (B) membrane-mimicking solution [50% (*v/v*) trifluoroethanol (TFE) in NH4Ac]. Figure S5. The estimated secondary structure contents analysed by BeStSel. The (A1) Brevinin-2GUb, (B1) tB2U, (C1) tB2U-α, (D1) tB2U-K, (E1) tB2U-6K, (F1) 7-tB2U-K, and (G1) 14-tB2U-K was in the environment of 20mM NH4Ac. The (A2) Brevinin-2GUb, (B2) tB2U, (C2) tB2U-α, (D2) tB2U-K, (E2) tB2U-6K, (F2) 7-tB2U-K and (G2) 14-tB2U-K was in the environment of 50% TFE in 20 mM NH4Ac. Table S1. Two-way ANOVA analysis of haemolysis activity of Brevinin-2GUb and its analogues. The significance was presented by the symbol **** (*p* < 0.0001). Table S2. Two-way ANOVA analysis of MTT assay of Brevinin-2GUb and its analogues against HaCaT. The significance was presented by the symbol **** (*p* < 0.0001). Table S3. The survival analysis of the antimicrobial activities in vivo of Brevinin-2GUb and its analogues. The significance was presented by the symbol * (*p* < 0.05), ** (*p* < 0.01), *** (*p* < 0.001) and **** (*p* < 0.0001).
