# Supplementary material for: Study on the Structure-Activity Relationship of an Antimicrobial Peptide, Brevinin-2GUb, from the Skin Secretion of Hylarana guentheri
Source: Antibiotics (Basel). 2021 Jul 22;10(8):895. doi: 10.3390/antibiotics10080895 (PMC8388802; doi:10.3390/antibiotics10080895)
Supplement: Supplementary file 1 [file antibiotics-10-00895-s001.zip › antibiotics-1306997-supplementary.pdf]

Supplementary Materials

Study on the Structure-activity Relationship of an Antimicrobial Peptide, Brevinin-2GUb, from the Skin Secretion of *Hylarana guntheri*

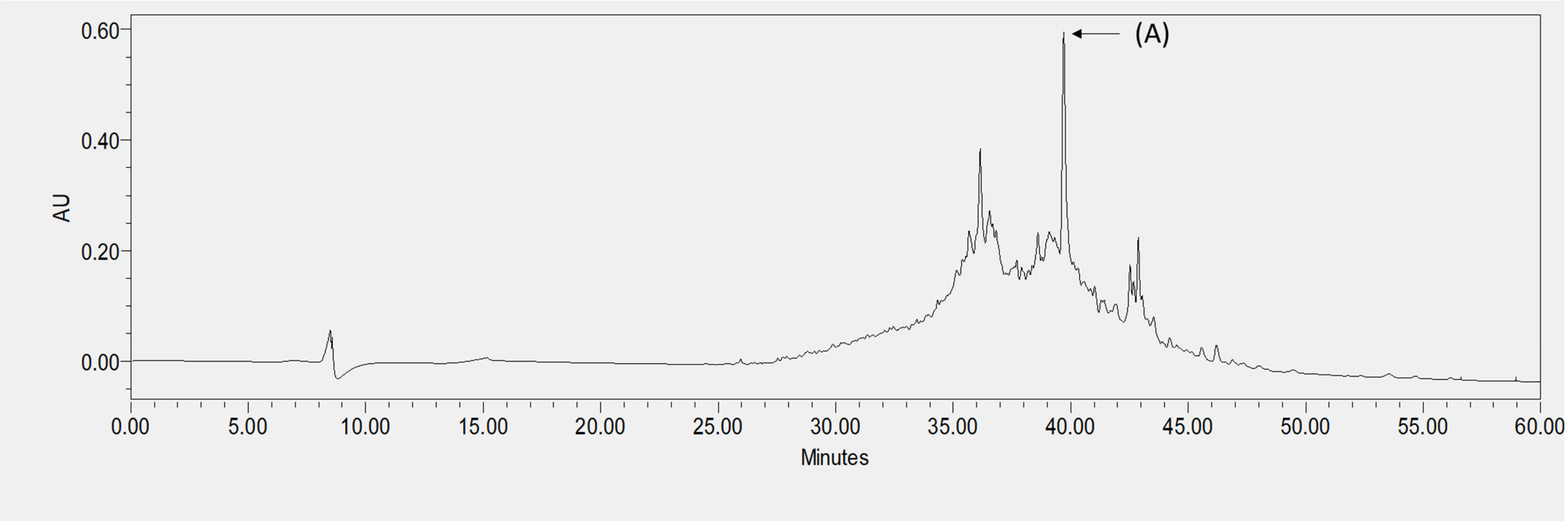

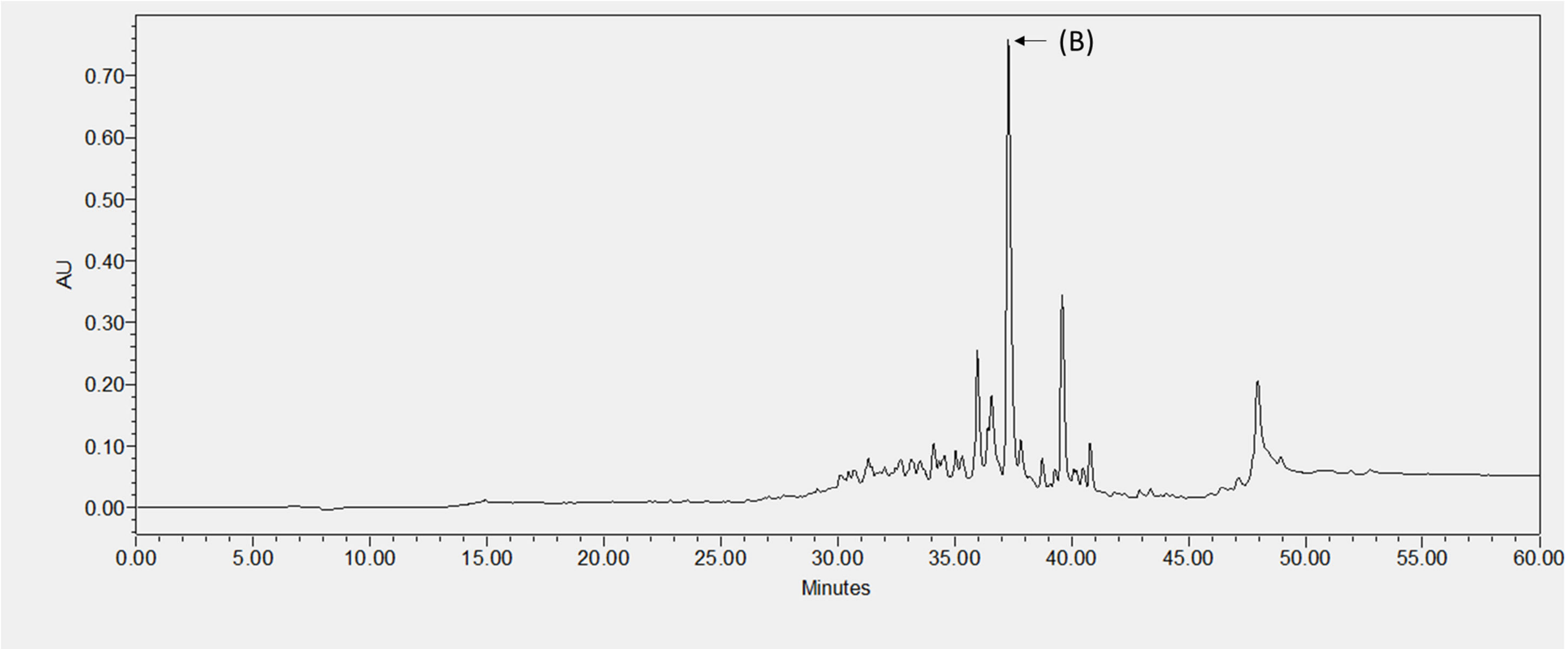

(B)
AU
0.70
0.60
0.50
0.40
0.30
0.20
0.10
0.00
0.00
5.00
10.00
15.00
20.00
25.00
30.00
35.00
40.00
45.00
50.00
55.00
60.00
Minutes

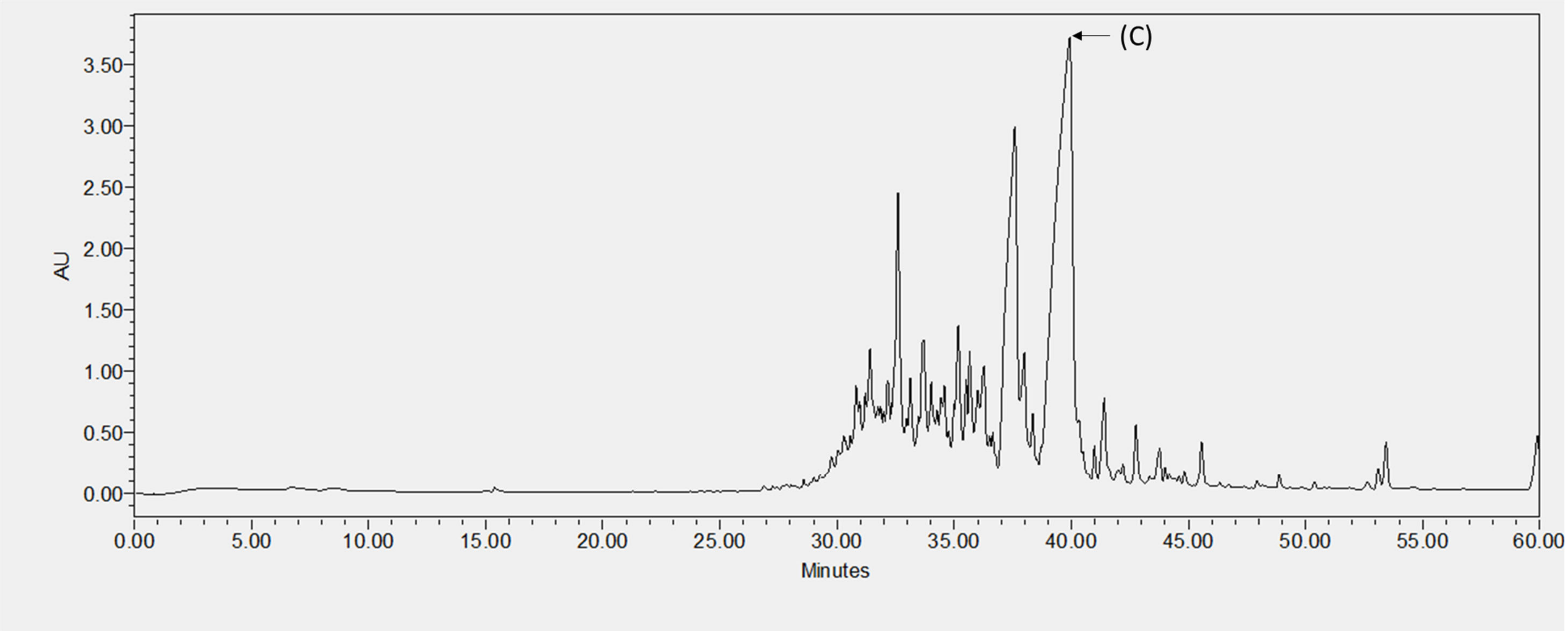

(C)
AU
3.50
3.00
2.50
2.00
1.50
1.00
0.50
0.00
0.00
5.00
10.00
15.00
20.00
25.00
30.00
35.00
40.00
45.00
50.00
55.00
60.00
Minutes

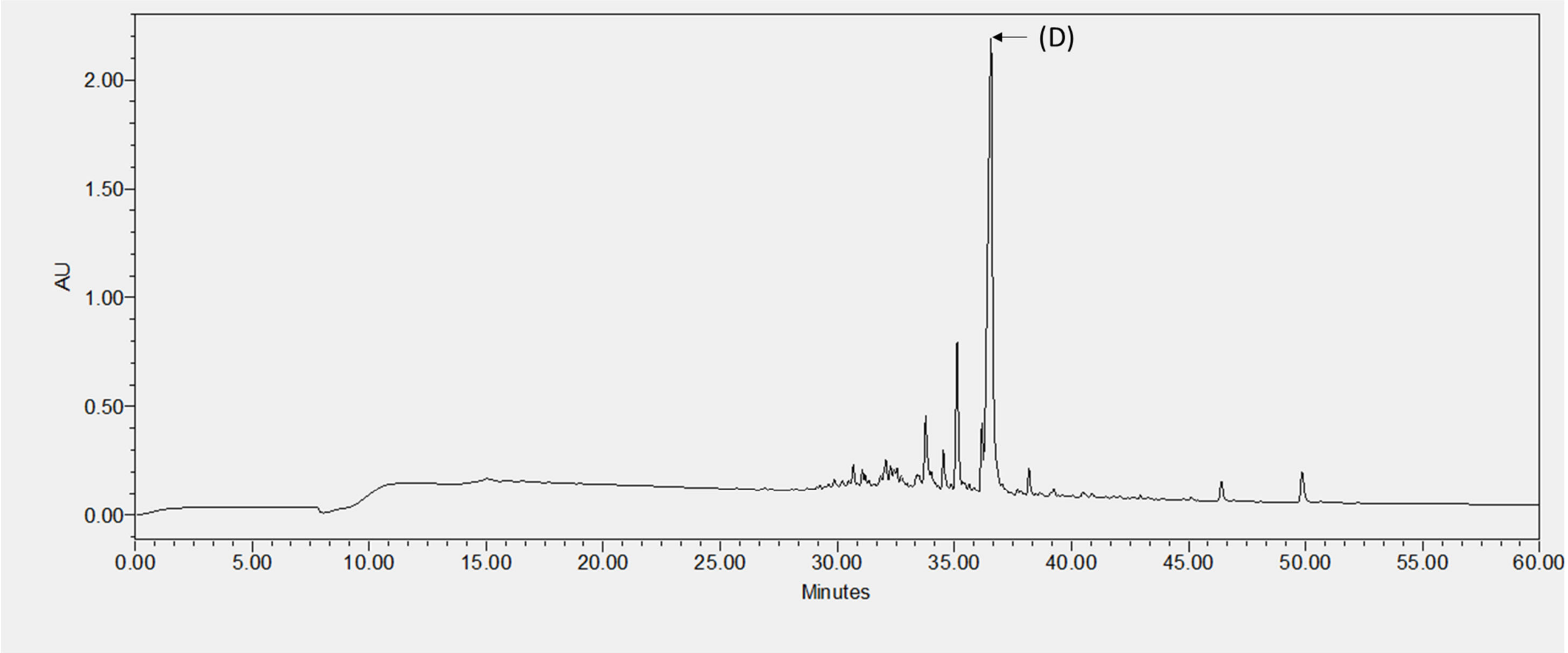

(D)
AU
2.00
1.50
1.00
0.50
0.00
0.00
5.00
10.00
15.00
20.00
25.00
30.00
35.00
40.00
45.00
50.00
55.00
60.00
Minutes

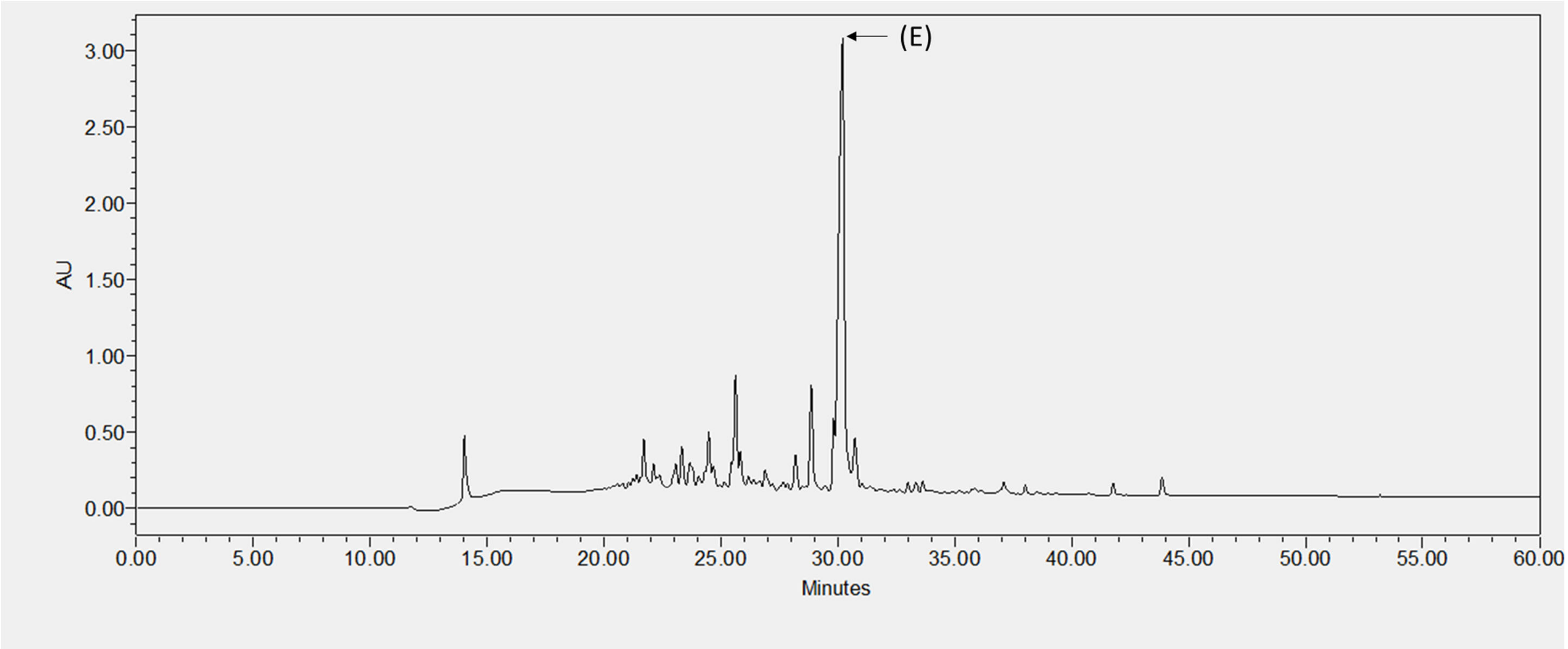
(E)
3.00
2.50
2.00
1.50
1.00
0.50
0.00
AU
0.00
5.00
10.00
15.00
20.00
25.00
30.00
35.00
40.00
45.00
50.00
55.00
60.00
Minutes

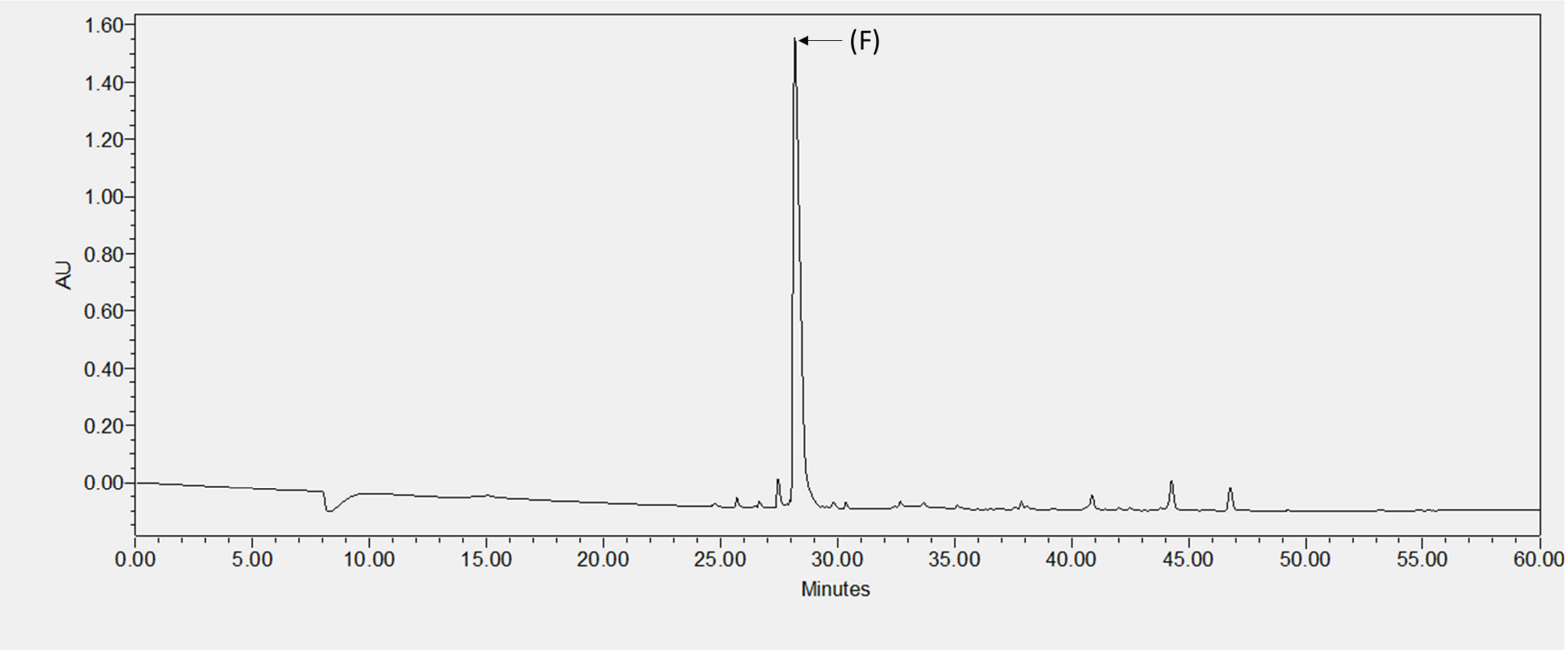

(F)
AU
Minutes
1.60
1.40
1.20
1.00
0.80
0.60
0.40
0.20
0.00
0.00
5.00
10.00
15.00
20.00
25.00
30.00
35.00
40.00
45.00
50.00
55.00
60.00

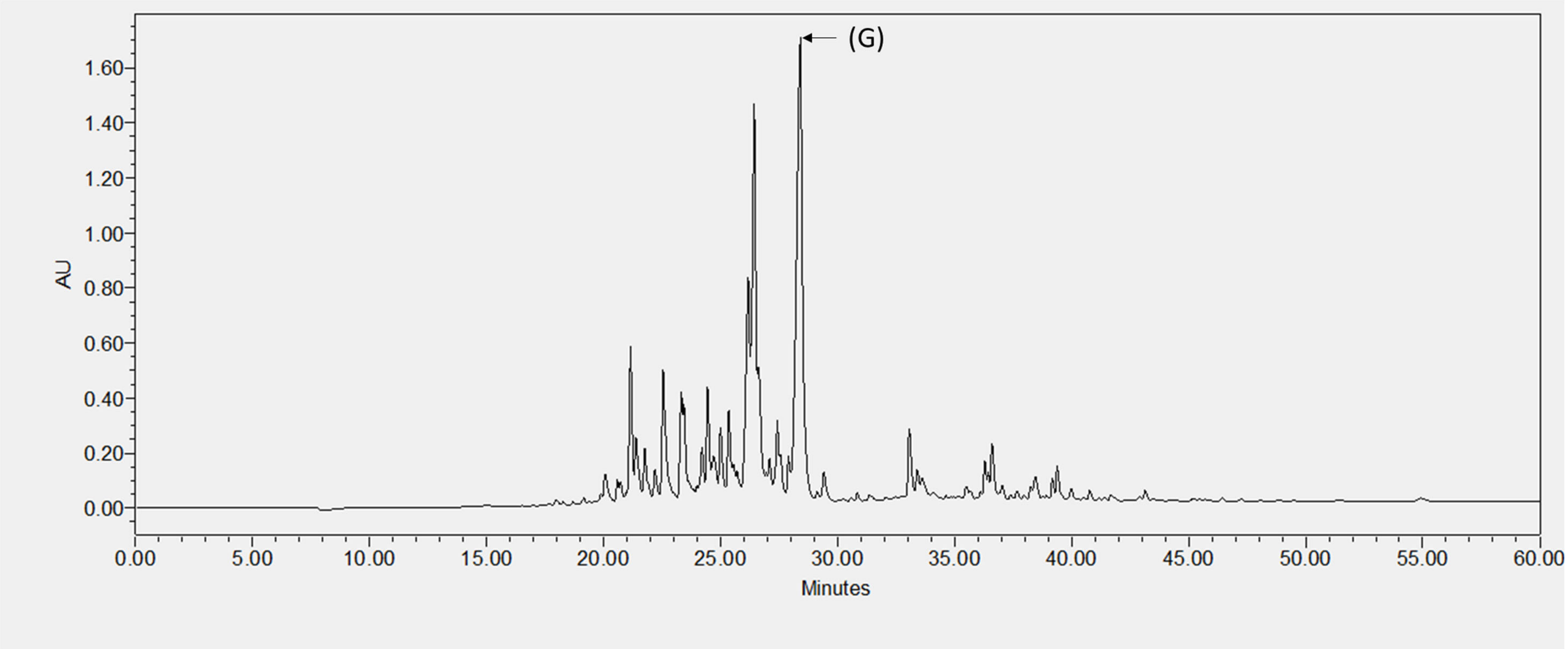

Figure S1. The RP-HPLC chromatograms of (A) Brevinin-2GUb, (B) tB2U, (C) tB2U-α, (D) tB2U-K, (E) tB2U-6K, (F) 7-tB2U-K and (G) 14-tB2U-K. The elution peak of each peptide is indicated by the arrow.

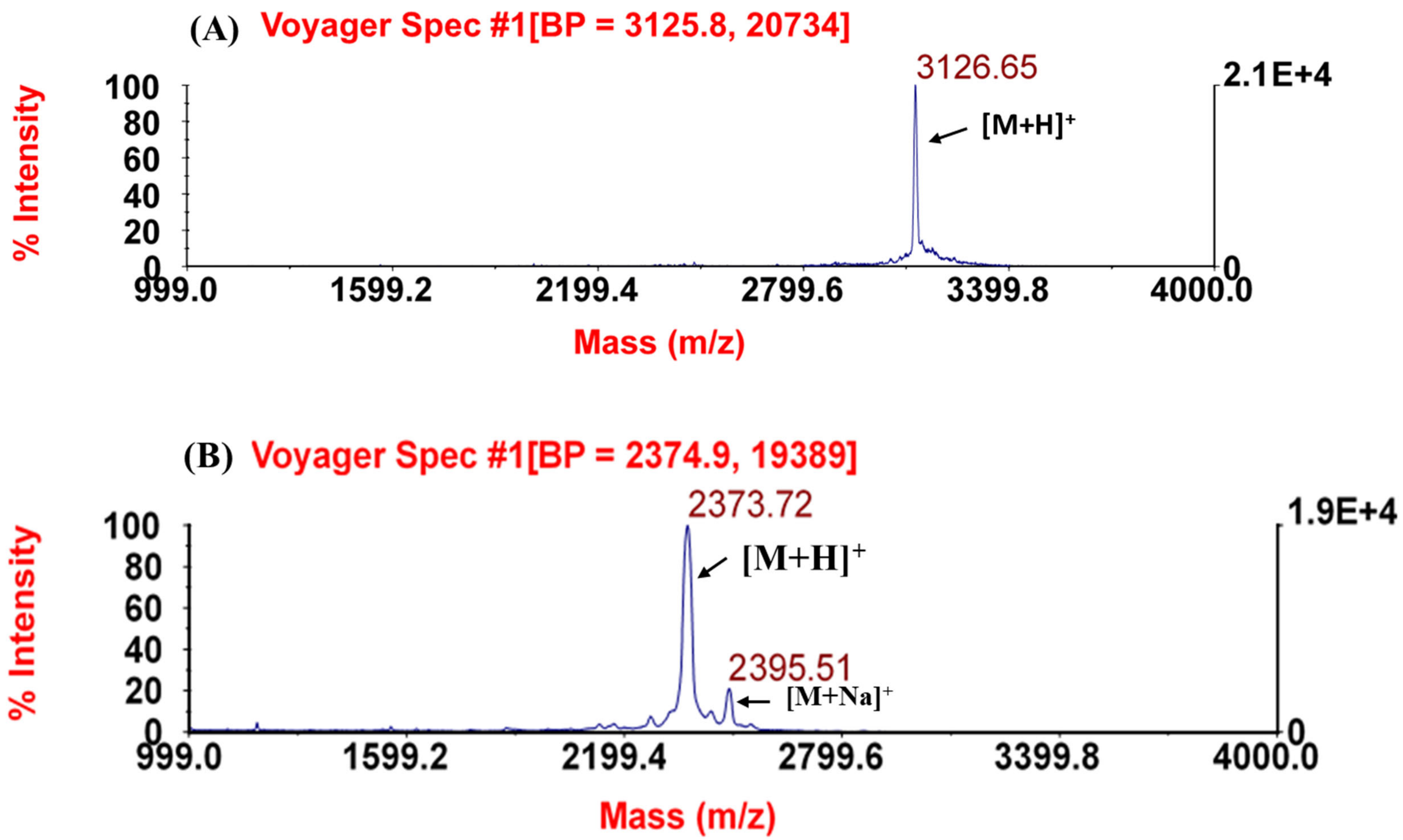

(A)
Voyager Spec #1[BP = 3125.8, 20734]
3126.65
[M+H]+
2.1E+4
% Intensity
100
80
60
40
20
0
999.0
1599.2
2199.4
2799.6
3399.8
4000.0
Mass (m/z)
(B)
Voyager Spec #1[BP = 2374.9, 19389]
2373.72
[M+H]+
1.9E+4
2395.51
[M+Na]+
% Intensity
Mass (m/z)

(C)

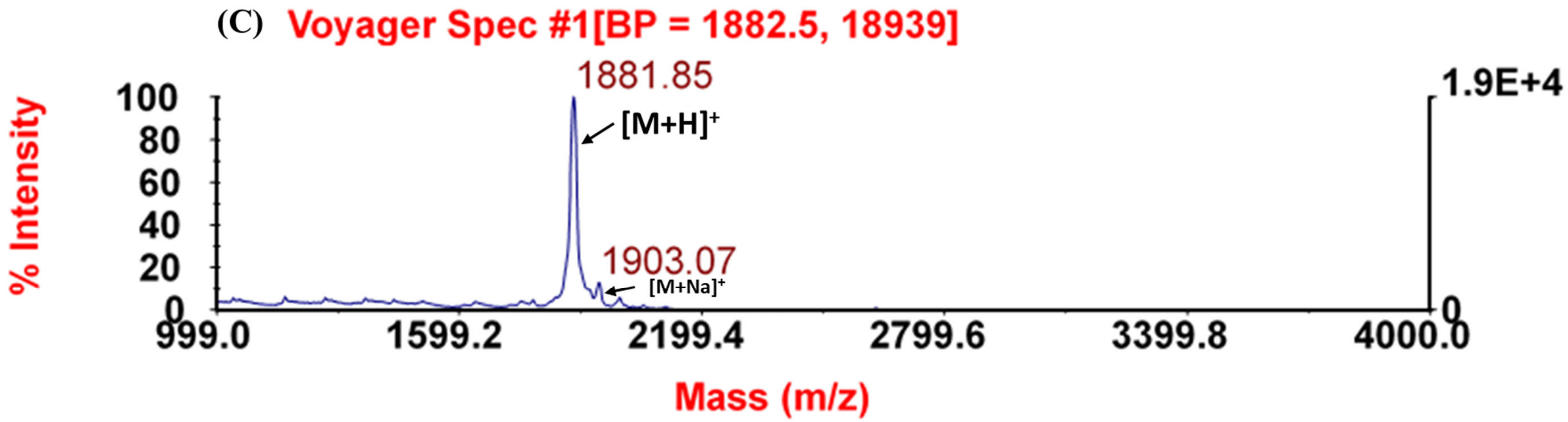
Voyager Spec #1[BP = 1882.5, 18939]
1881.85
[M+H]+
1903.07
[M+Na]+
1.9E+4
% Intensity
100
80
60
40
20
0
999.0
1599.2
2199.4
2799.6
3399.8
4000.0
Mass (m/z)

(D)

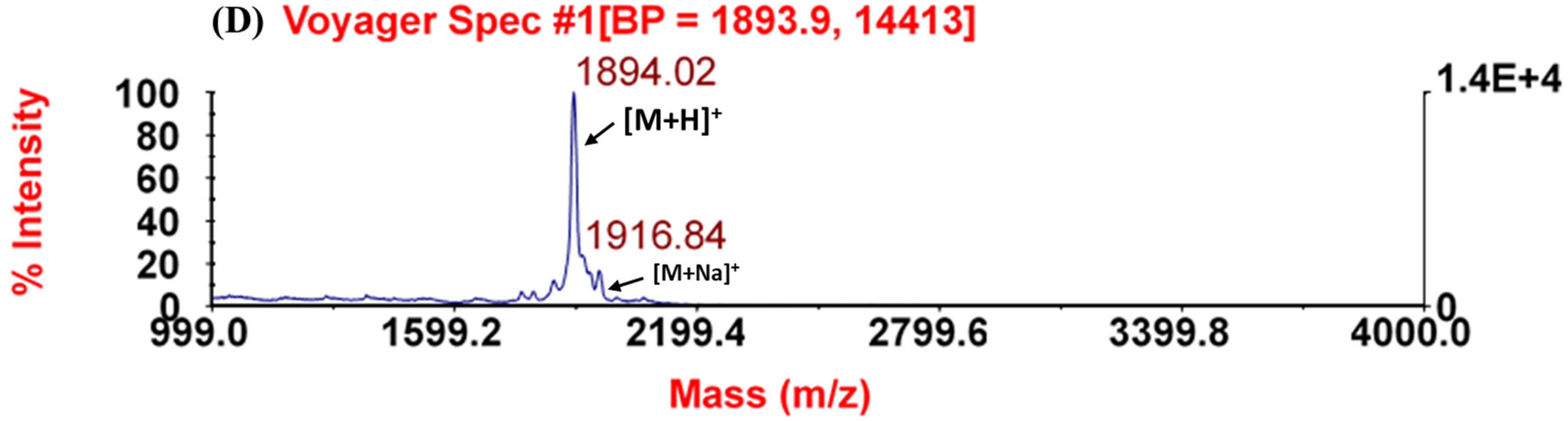
Voyager Spec #1[BP = 1893.9, 14413]
1894.02
[M+H]+
1916.84
[M+Na]+
1.4E+4
% Intensity
100
80
60
40
20
0
999.0
1599.2
2199.4
2799.6
3399.8
4000.0
Mass (m/z)

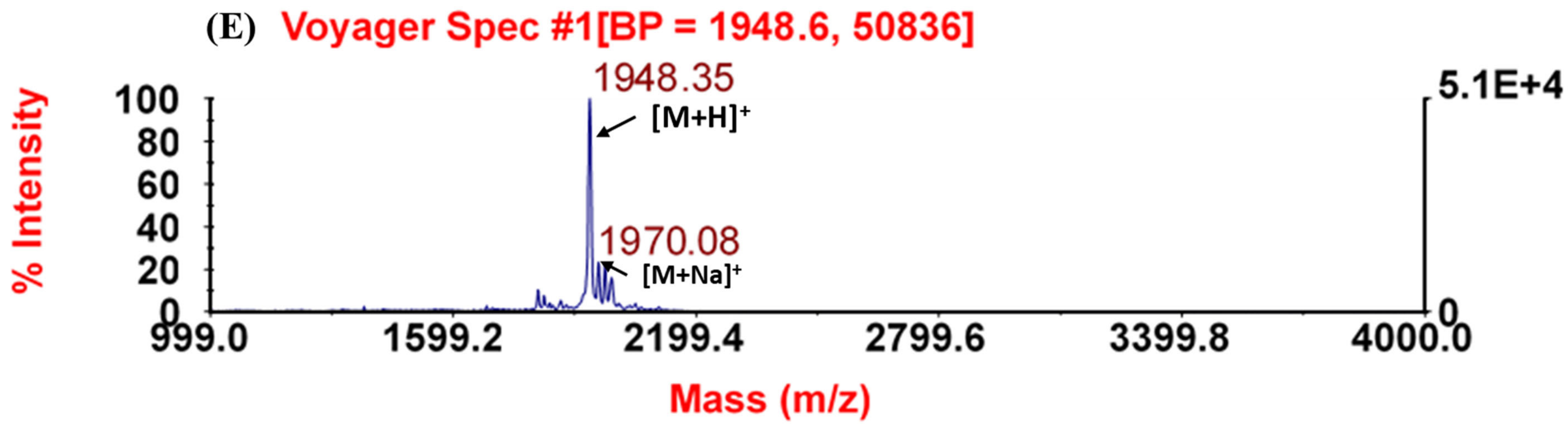
(E) Voyager Spec #1[BP = 1948.6, 50836]
1948.35
[M+H]+
1970.08
[M+Na]+
% Intensity
100
80
60
40
20
0
5.1E+4
0
999.0
1599.2
2199.4
2799.6
3399.8
4000.0
Mass (m/z)

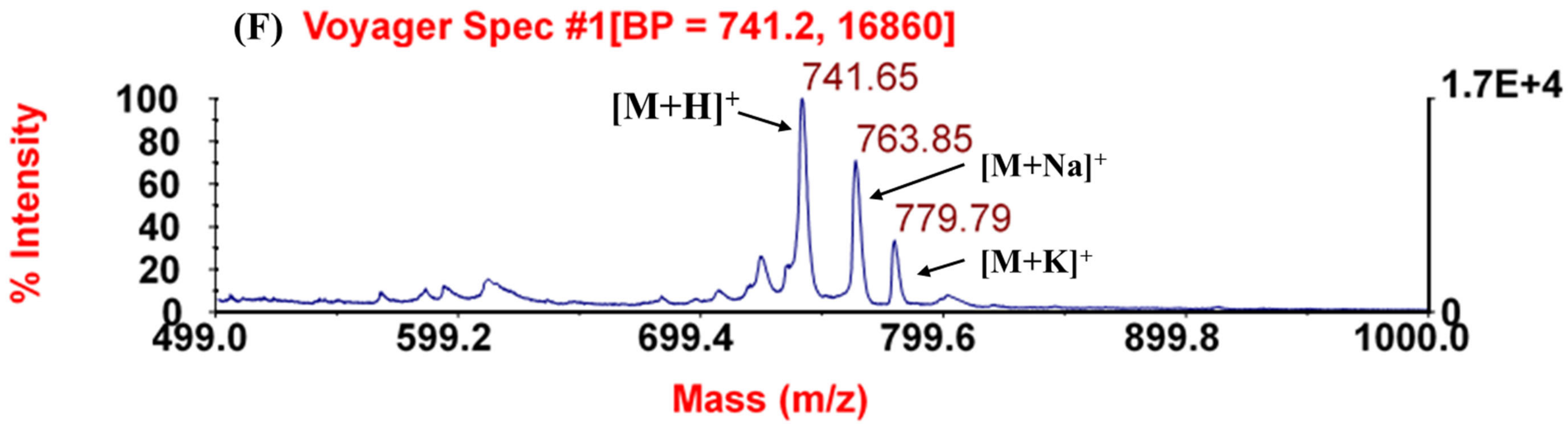
(F) Voyager Spec #1[BP = 741.2, 16860]
741.65
[M+H]+
763.85
[M+Na]+
779.79
[M+K]+
% Intensity
100
80
60
40
20
0
1.7E+4
0
499.0
599.2
699.4
799.6
899.8
1000.0
Mass (m/z)

(G)

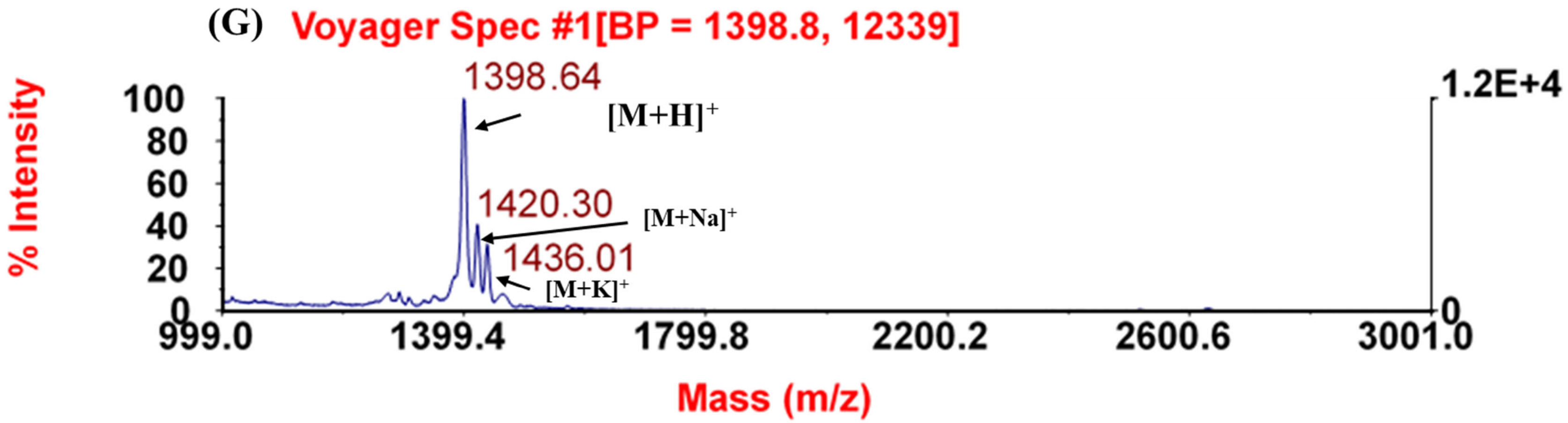

Figure S2. The mass spectra of (A) Brevinin-2GUb, (B) tB2U, (C) tB2U-α, (D) tB2U-K, (E) tB2U-6K, (F) 7-tB2U-K and (G) 14-tB2U-K obtained from MALDI-TOF MS. The observed $[M+H]^+$ ion, the sodium and potassium adduct ions are indicated by arrows.

(A)

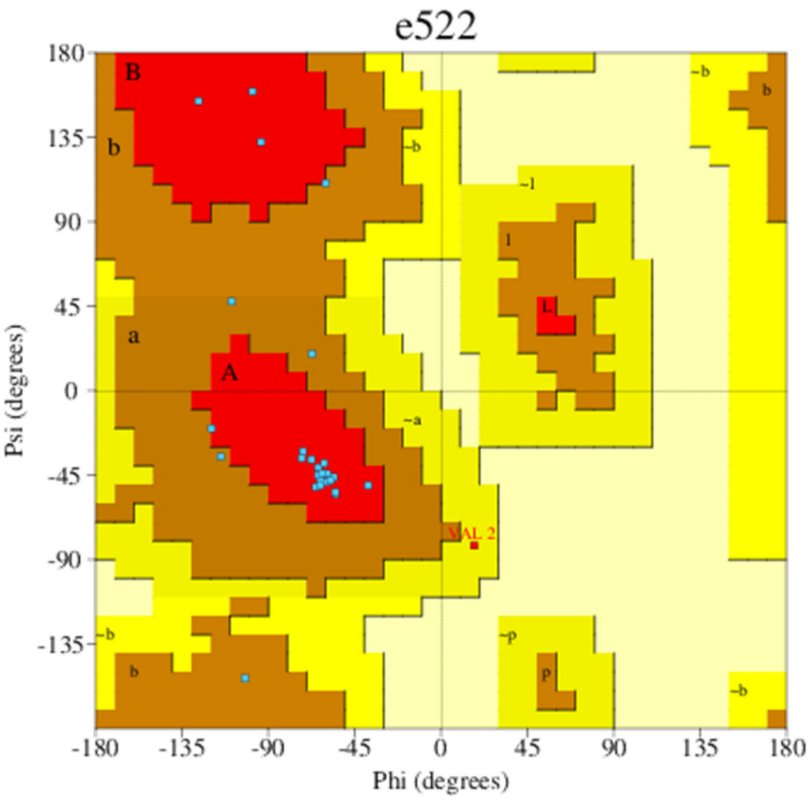
e522
Psi (degrees)
Phi (degrees)
180
135
90
45
0
-45
-90
-135
-180
-135
-90
-45
0
45
90
135
180
B
b
a
A
~b
~l
l
L
~a
~p
p
VAL 2

(B)

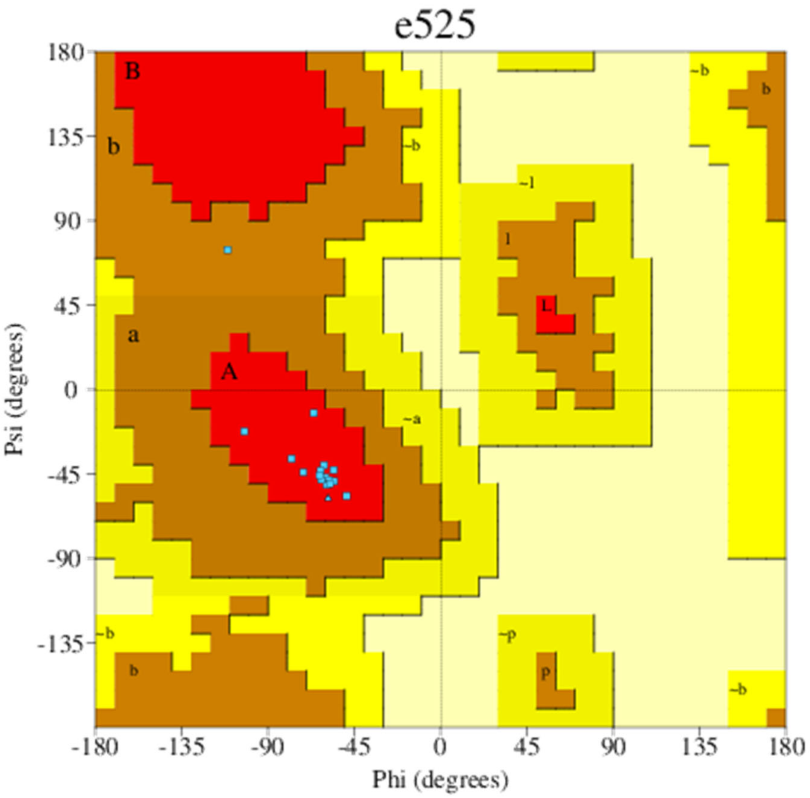
e525
Psi (degrees)
Phi (degrees)
180
135
90
45
0
-45
-90
-135
-180
-135
-90
-45
0
45
90
135
180
B
b
a
A
~b
~l
l
L
~a
~p
p

(C)

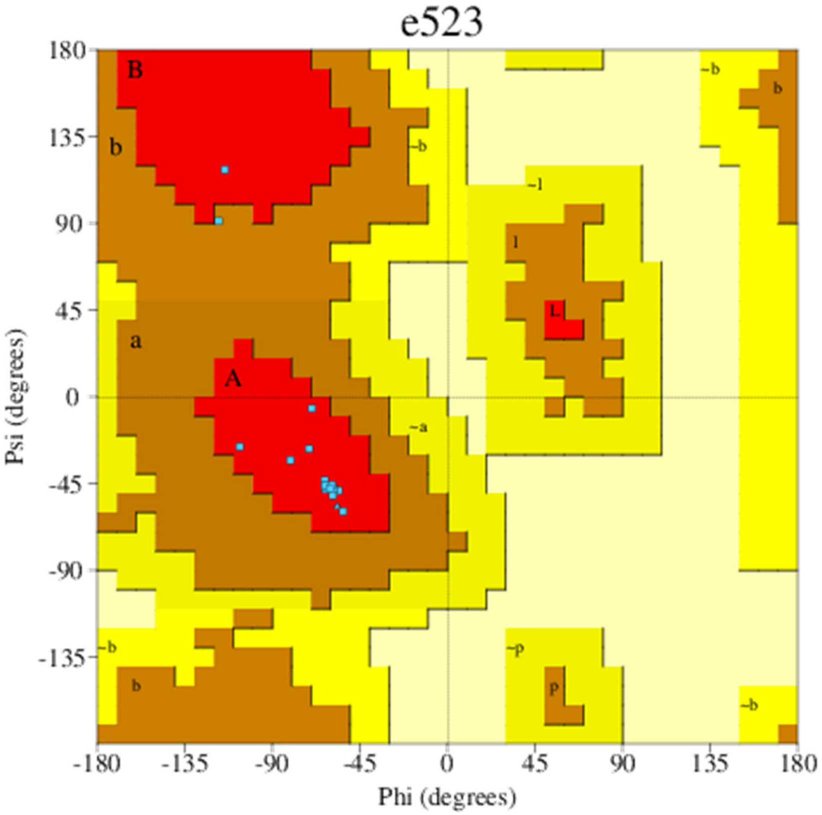
e523
Psi (degrees)
Phi (degrees)
180
135
90
45
0
-45
-90
-135
-180
-135
-90
-45
0
45
90
135
180
B
b
~b
~l
l
L
a
A
~a
~p
p
b
~b

(D)

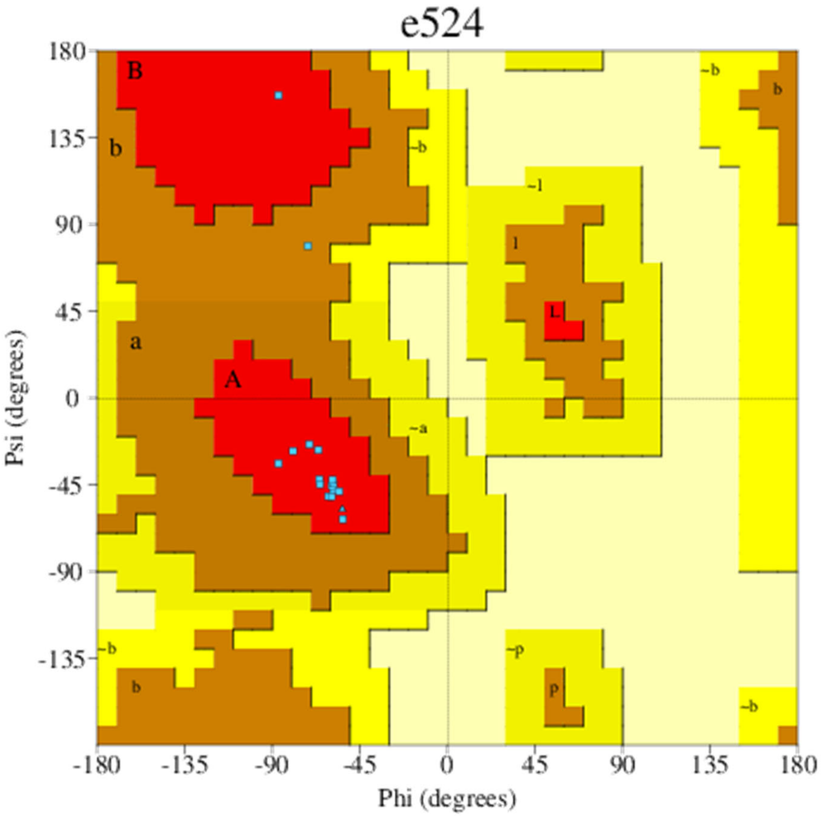
e524
Psi (degrees)
Phi (degrees)
180
135
90
45
0
-45
-90
-135
-180
-135
-90
-45
0
45
90
135
180
B
b
~b
~l
l
L
a
A
~a
~p
p
b
~b

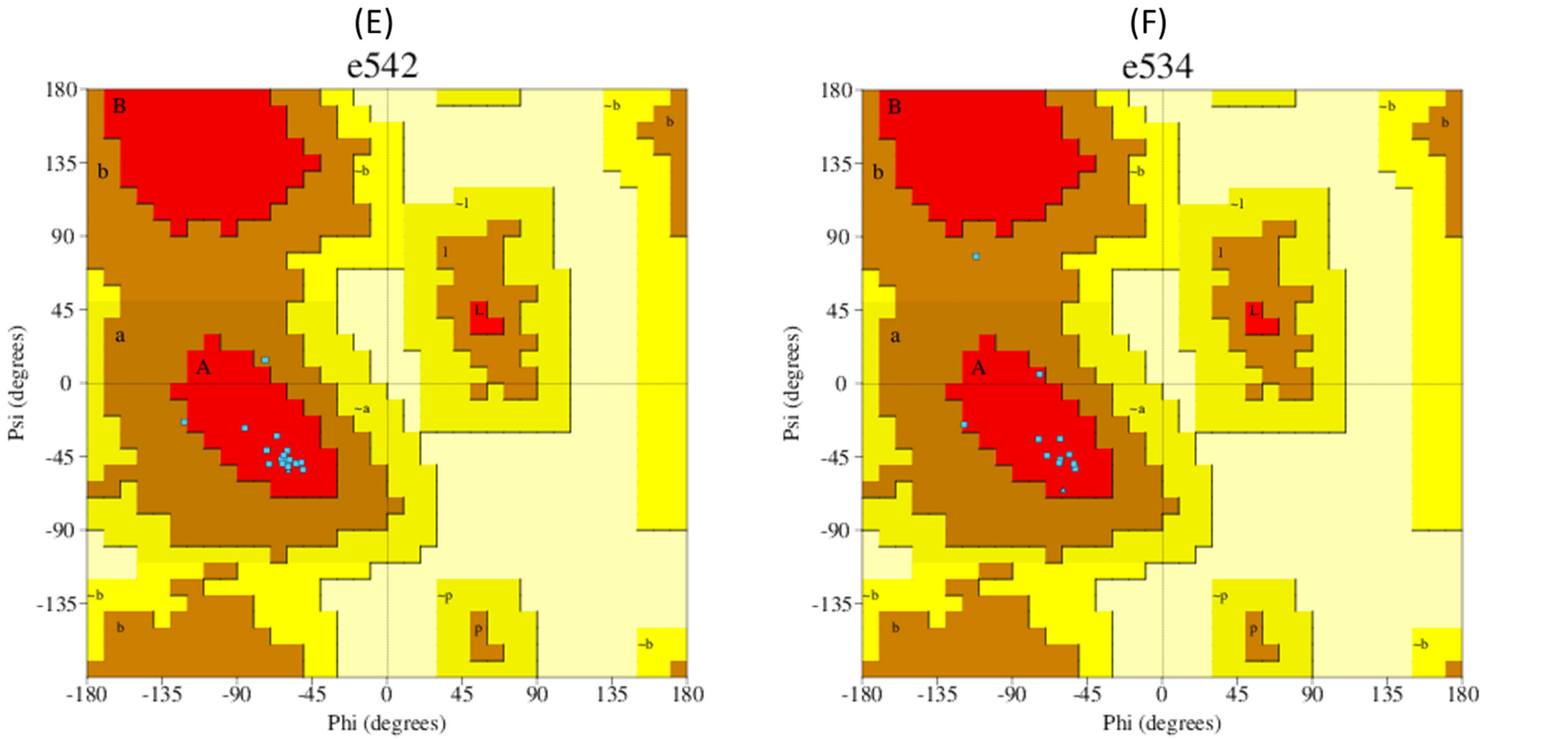

Figure S3. The Ramachandran plots of (A) Brevinin-2GUb, (B) tB2U, (C) tB2U-α, (D) tB2U-K, (E) tB2U-6K and (F) 14-tB2U-K predicted by PROCHECK (https://www.ebi.ac.uk/thornton-srv/software/PROCHECK/). The X and Y axis is the torsional angles-Psi (Ψ) and Phi (Φ),

respectively. The Glycine residues are separately represented by triangles and the red regions are the core areas which represent the most favourable combinations of phi-psi values.

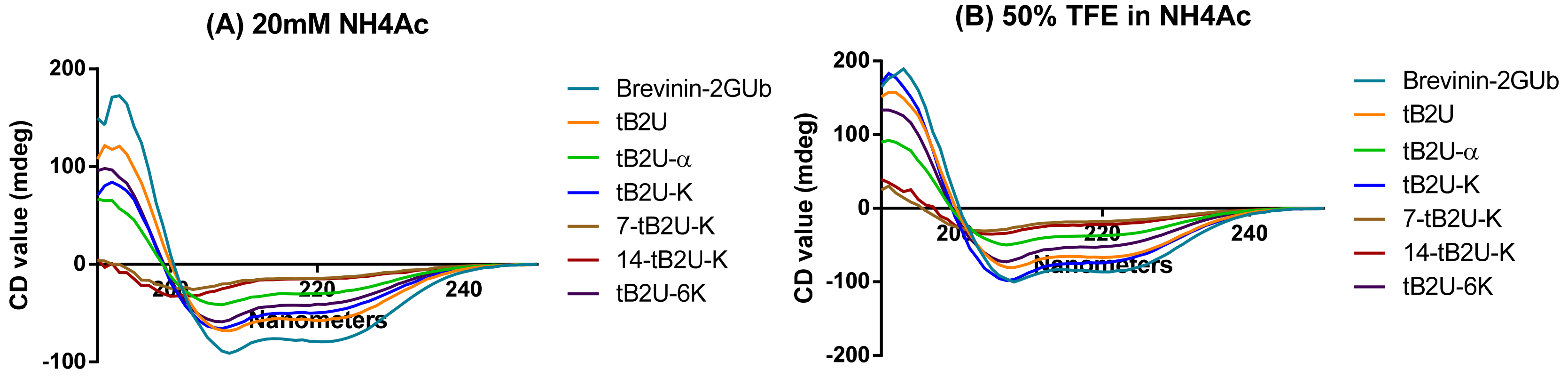

Figure S4. The CD spectra of Brevinin-2GUb and its analogues in (A) aqueous solution (20mM NH_4Ac) and (B) membrane-mimicking solution [50% (v/v) trifluoroethanol (TFE) in NH_4Ac].

(A1)

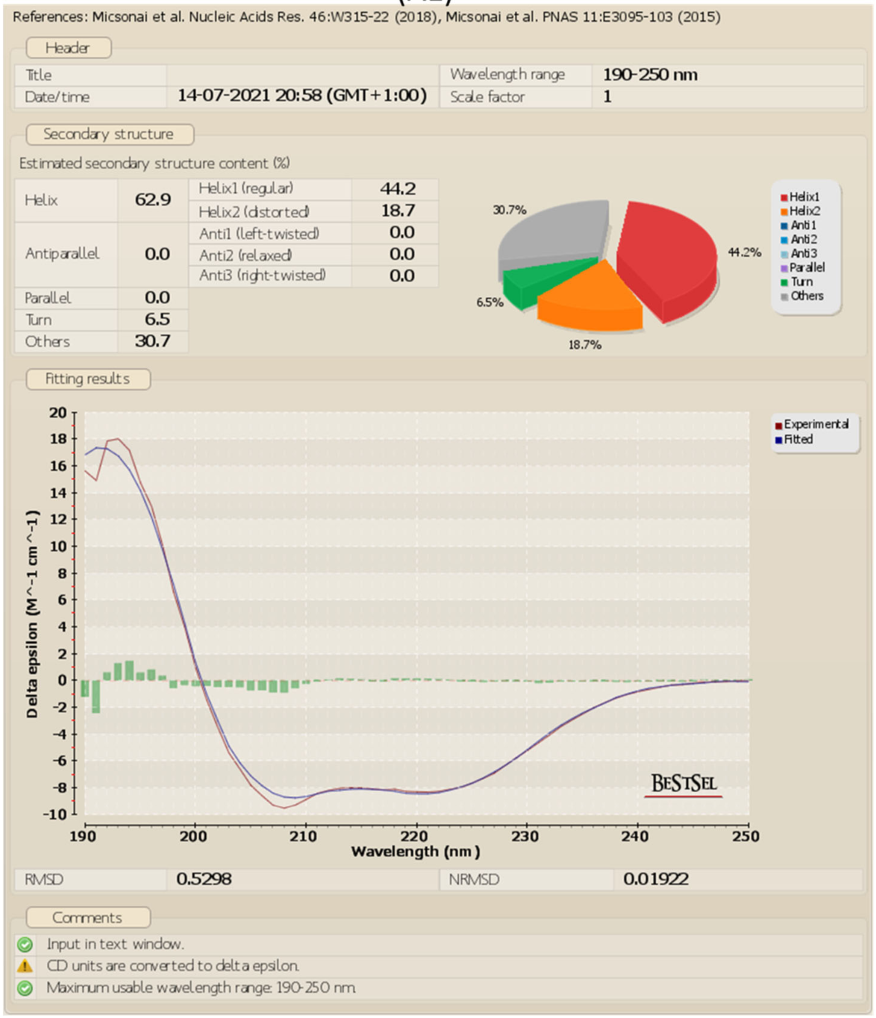
References: Micsonai et al. Nucleic Acids Res. 46:W315-22 (2018), Micsonai et al. PNAS 11:E3095-103 (2015)
Header
Title
Date/time
14-07-2021 20:58 (GMT+1:00)
Wavelength range
190-250 nm
Scale factor
1
Secondary structure
Estimated secondary structure content (%)
Helix
62.9
Helix1 (regular)
44.2
Helix2 (distorted)
18.7
Antiparallel
0.0
Anti1 (left-twisted)
0.0
Anti2 (relaxed)
0.0
Anti3 (right-twisted)
0.0
Parallel
0.0
Turn
6.5
Others
30.7
30.7%
44.2%
6.5%
18.7%
Helix1
Helix2
Anti1
Anti2
Anti3
Parallel
Turn
Others
Fitting results
Delta epsilon (M^-1 cm^-1)
Wavelength (nm)
Experimental
Fitted
BeStSel
RMSD
0.5298
NRMSD
0.01922
Comments
Input in text window.
CD units are converted to delta epsilon.
Maximum usable wavelength range: 190-250 nm.

(A2)

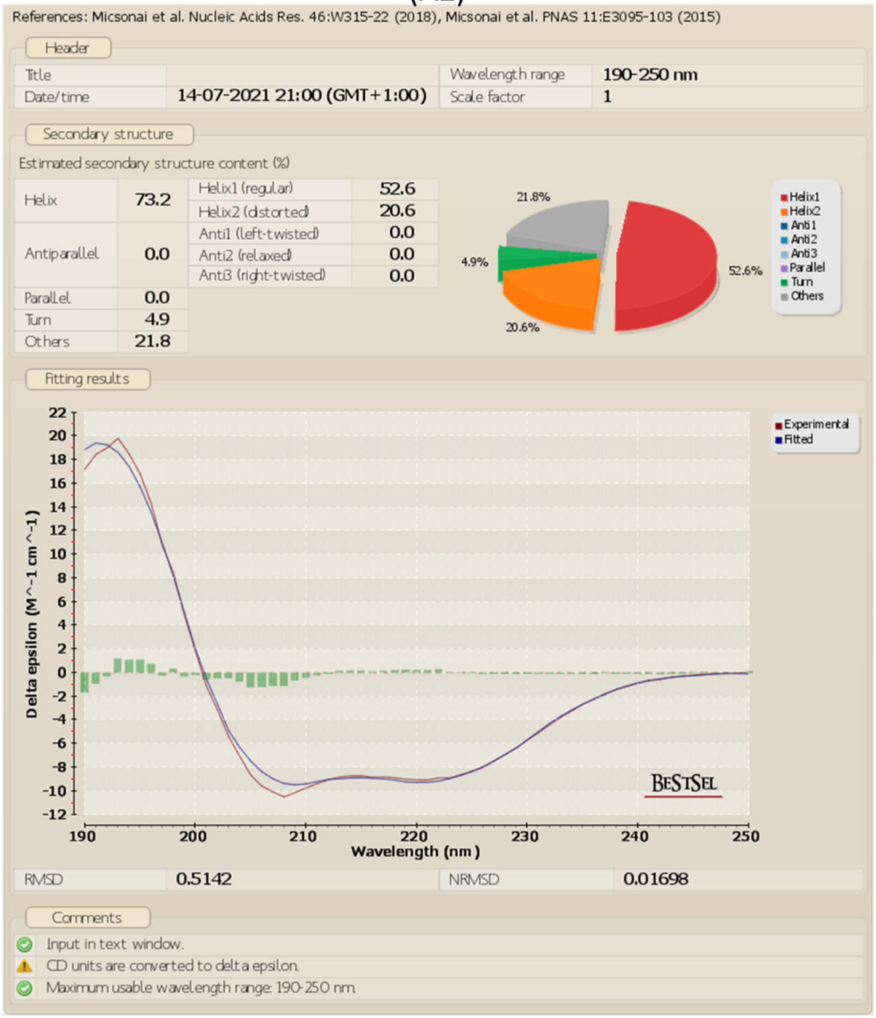
References: Micsonai et al. Nucleic Acids Res. 46:W315-22 (2018), Micsonai et al. PNAS 11:E3095-103 (2015)
Header
Title
Date/time
14-07-2021 21:00 (GMT+1:00)
Wavelength range
190-250 nm
Scale factor
1
Secondary structure
Estimated secondary structure content (%)
Helix
73.2
Helix1 (regular)
52.6
Helix2 (distorted)
20.6
Antiparallel
0.0
Anti1 (left-twisted)
0.0
Anti2 (relaxed)
0.0
Anti3 (right-twisted)
0.0
Parallel
0.0
Turn
4.9
Others
21.8
21.8%
4.9%
52.6%
20.6%
Helix1
Helix2
Anti1
Anti2
Anti3
Parallel
Turn
Others
Fitting results
Delta epsilon (M^-1 cm^-1)
Wavelength (nm)
Experimental
Fitted
BeStSel
RMSD
0.5142
NRMSD
0.01698
Comments
Input in text window.
CD units are converted to delta epsilon.
Maximum usable wavelength range: 190-250 nm.

(B1)

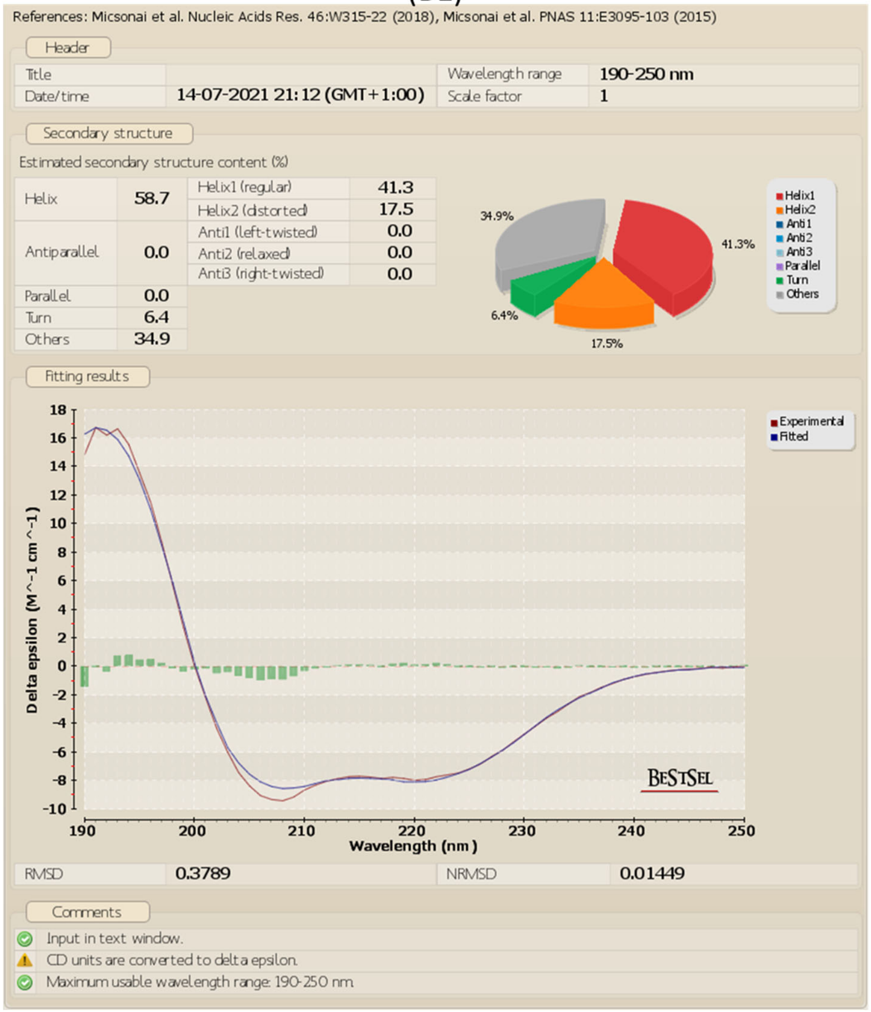
References: Micsonai et al. Nucleic Acids Res. 46:W315-22 (2018), Micsonai et al. PNAS 11:E3095-103 (2015)
Header
Title
Wavelength range
190-250 nm
Date/time
14-07-2021 21:12 (GMT+1:00)
Scale factor
1
Secondary structure
Estimated secondary structure content (%)
Helix 58.7
Helix1 (regular) 41.3
Helix2 (distorted) 17.5
Antiparallel 0.0
Anti1 (left-twisted) 0.0
Anti2 (relaxed) 0.0
Anti3 (right-twisted) 0.0
Parallel 0.0
Turn 6.4
Others 34.9
34.9%
41.3%
6.4%
17.5%
Helix1
Helix2
Anti1
Anti2
Anti3
Parallel
Turn
Others
Fitting results
Experimental
Fitted
Delta epsilon (M^-1 cm^-1)
Wavelength (nm)
BeStSel
RMSD 0.3789
NRMSD 0.01449
Comments
Input in text window.
CD units are converted to delta epsilon.
Maximum usable wavelength range: 190-250 nm.

(B2)

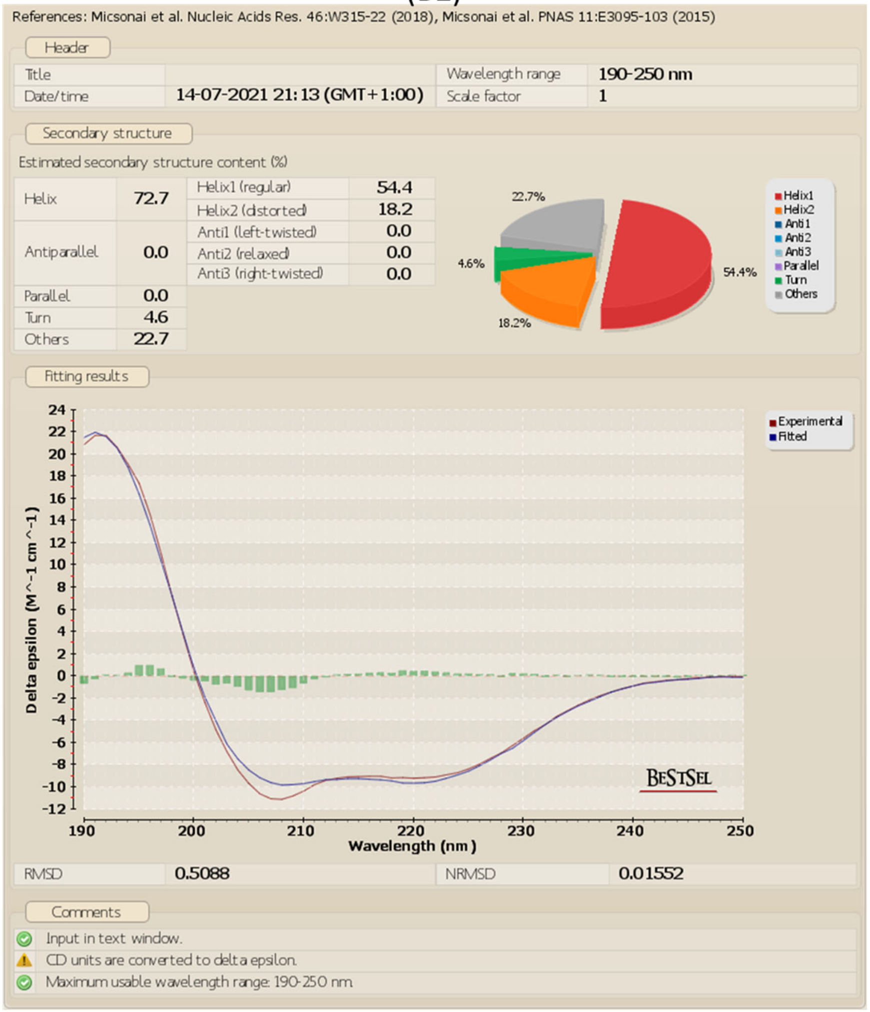
References: Micsonai et al. Nucleic Acids Res. 46:W315-22 (2018), Micsonai et al. PNAS 11:E3095-103 (2015)
Header
Title
Wavelength range
190-250 nm
Date/time
14-07-2021 21:13 (GMT+1:00)
Scale factor
1
Secondary structure
Estimated secondary structure content (%)
Helix 72.7
Helix1 (regular) 54.4
Helix2 (distorted) 18.2
Antiparallel 0.0
Anti1 (left-twisted) 0.0
Anti2 (relaxed) 0.0
Anti3 (right-twisted) 0.0
Parallel 0.0
Turn 4.6
Others 22.7
22.7%
4.6%
54.4%
18.2%
Helix1
Helix2
Anti1
Anti2
Anti3
Parallel
Turn
Others
Fitting results
Experimental
Fitted
Delta epsilon (M^-1 cm^-1)
Wavelength (nm)
BeStSel
RMSD 0.5088
NRMSD 0.01552
Comments
Input in text window.
CD units are converted to delta epsilon.
Maximum usable wavelength range: 190-250 nm.

(C1)

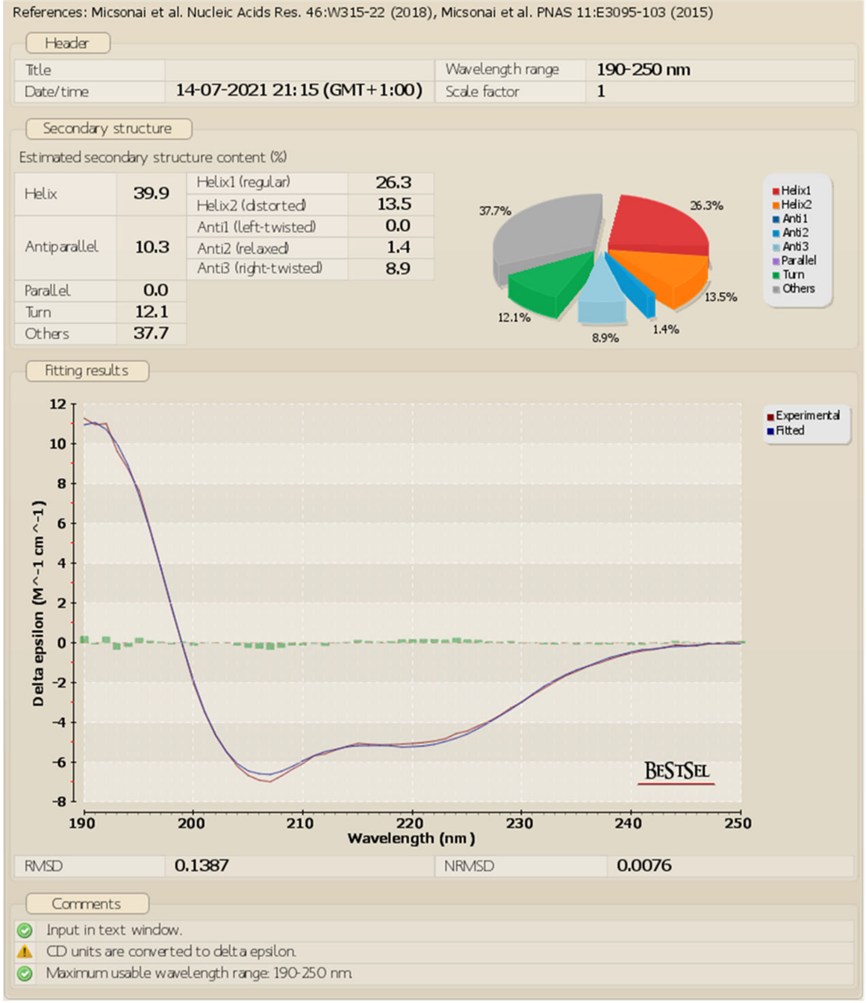
References: Micsonai et al. Nucleic Acids Res. 46:W315-22 (2018), Micsonai et al. PNAS 11:E3095-103 (2015)
Header
Title
Date/time 14-07-2021 21:15 (GMT+1:00)
Wavelength range 190-250 nm
Scale factor 1
Secondary structure
Estimated secondary structure content (%)
Helix 39.9
Helix1 (regular) 26.3
Helix2 (distorted) 13.5
Antiparallel 10.3
Anti1 (left-twisted) 0.0
Anti2 (relaxed) 1.4
Anti3 (right-twisted) 8.9
Parallel 0.0
Turn 12.1
Others 37.7
37.7%
26.3%
13.5%
12.1%
8.9%
1.4%
Helix1
Helix2
Anti1
Anti2
Anti3
Parallel
Turn
Others
Fitting results
Delta epsilon (M^-1 cm^-1)
Wavelength (nm)
Experimental
Fitted
BeStSel
RMSD 0.1387
NRMSD 0.0076
Comments
Input in text window.
CD units are converted to delta epsilon.
Maximum usable wavelength range: 190-250 nm.

(C2)

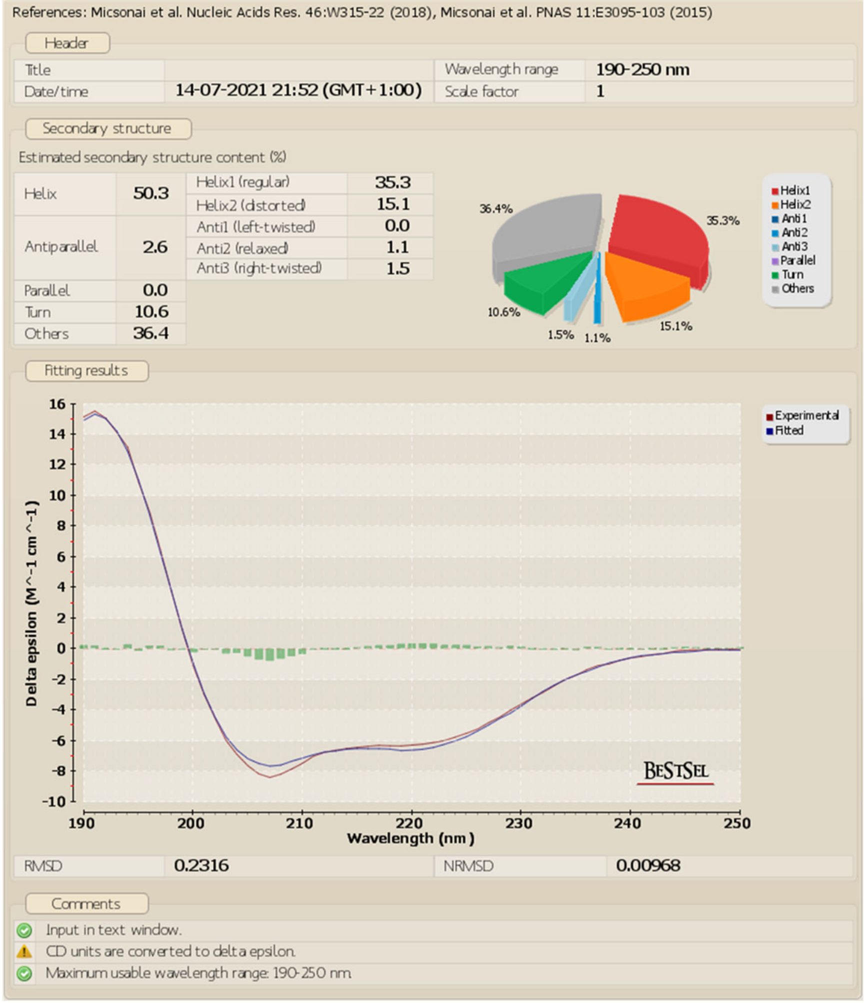
References: Micsonai et al. Nucleic Acids Res. 46:W315-22 (2018), Micsonai et al. PNAS 11:E3095-103 (2015)
Header
Title
Date/time 14-07-2021 21:52 (GMT+1:00)
Wavelength range 190-250 nm
Scale factor 1
Secondary structure
Estimated secondary structure content (%)
Helix 50.3
Helix1 (regular) 35.3
Helix2 (distorted) 15.1
Antiparallel 2.6
Anti1 (left-twisted) 0.0
Anti2 (relaxed) 1.1
Anti3 (right-twisted) 1.5
Parallel 0.0
Turn 10.6
Others 36.4
36.4%
35.3%
15.1%
10.6%
1.5%
1.1%
Helix1
Helix2
Anti1
Anti2
Anti3
Parallel
Turn
Others
Fitting results
Delta epsilon (M^-1 cm^-1)
Wavelength (nm)
Experimental
Fitted
BeStSel
RMSD 0.2316
NRMSD 0.00968
Comments
Input in text window.
CD units are converted to delta epsilon.
Maximum usable wavelength range: 190-250 nm.

(D1)

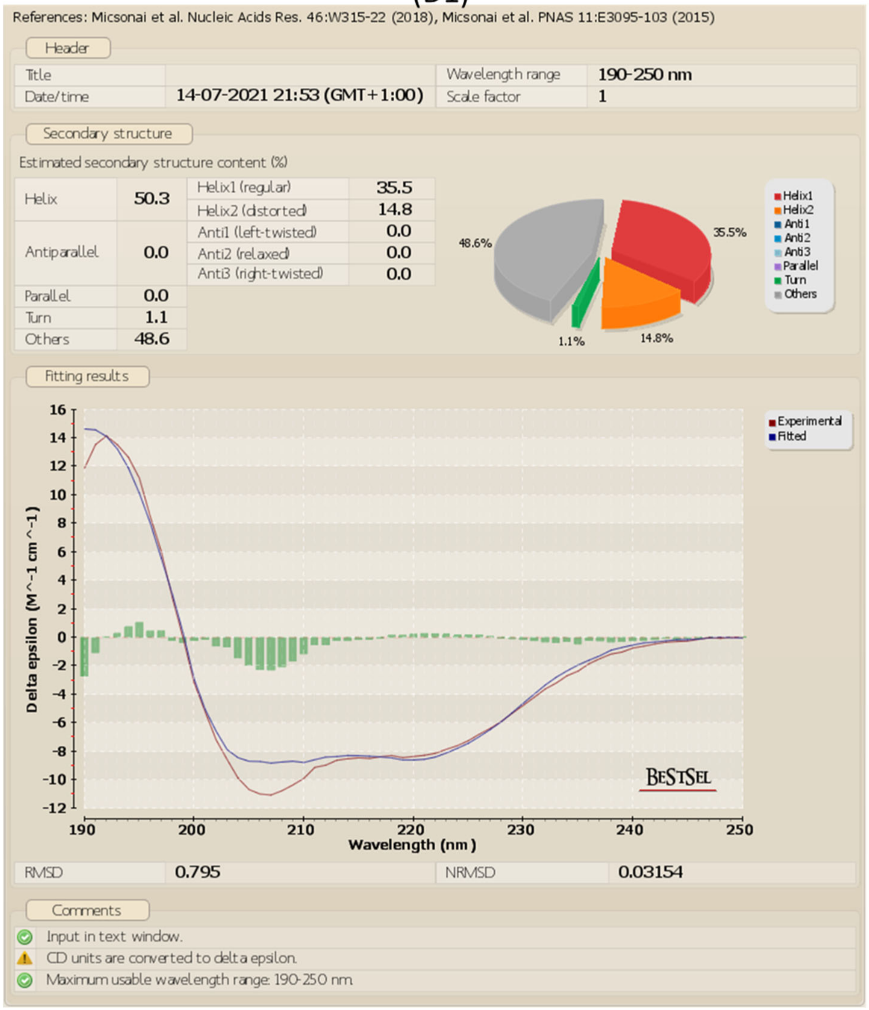
References: Micsonai et al. Nucleic Acids Res. 46:W315-22 (2018), Micsonai et al. PNAS 11:E3095-103 (2015)
Header
Title
Wavelength range
190-250 nm
Date/time
14-07-2021 21:53 (GMT+1:00)
Scale factor
1
Secondary structure
Estimated secondary structure content (%)
Helix 50.3
Helix1 (regular) 35.5
Helix2 (distorted) 14.8
Antiparallel 0.0
Anti1 (left-twisted) 0.0
Anti2 (relaxed) 0.0
Anti3 (right-twisted) 0.0
Parallel 0.0
Turn 1.1
Others 48.6
48.6%
35.5%
1.1%
14.8%
Helix1
Helix2
Anti1
Anti2
Anti3
Parallel
Turn
Others
Fitting results
Delta epsilon (M^-1 cm ^-1)
Wavelength (nm)
Experimental
Fitted
BESTSEL
RMSD 0.795
NRMSD 0.03154
Comments
Input in text window.
CD units are converted to delta epsilon.
Maximum usable wavelength range: 190-250 nm.

(D2)

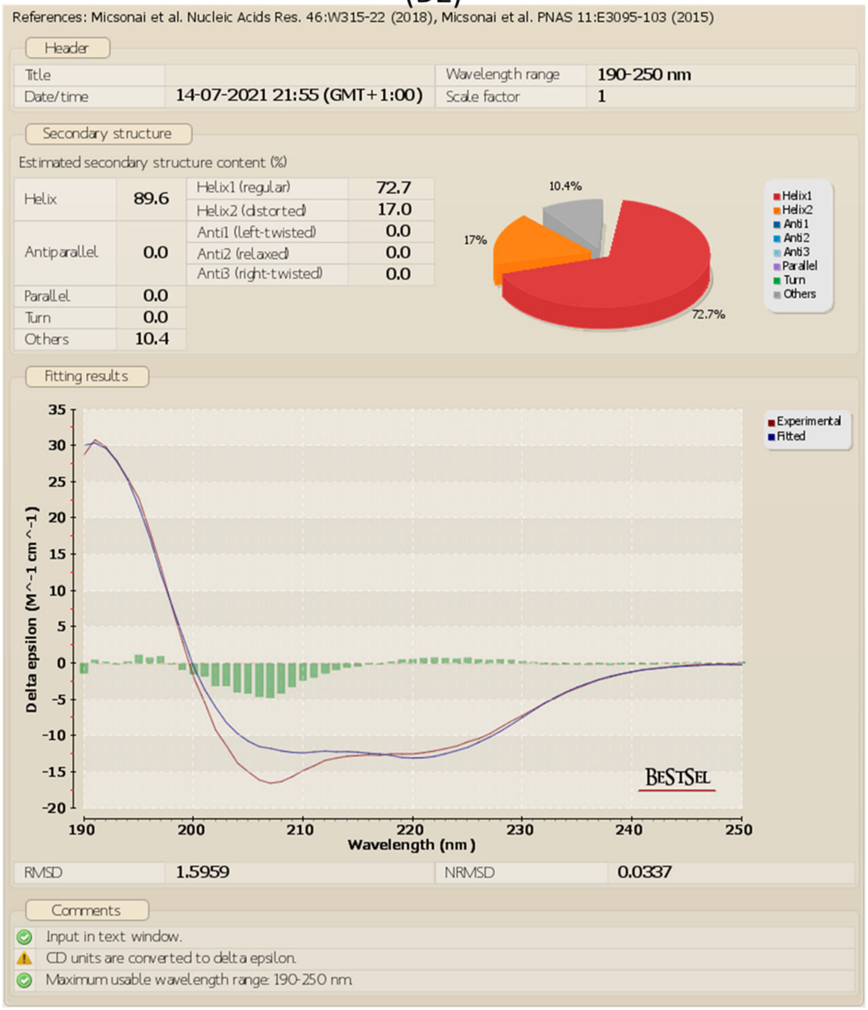
References: Micsonai et al. Nucleic Acids Res. 46:W315-22 (2018), Micsonai et al. PNAS 11:E3095-103 (2015)
Header
Title
Wavelength range
190-250 nm
Date/time
14-07-2021 21:55 (GMT+1:00)
Scale factor
1
Secondary structure
Estimated secondary structure content (%)
Helix 89.6
Helix1 (regular) 72.7
Helix2 (distorted) 17.0
Antiparallel 0.0
Anti1 (left-twisted) 0.0
Anti2 (relaxed) 0.0
Anti3 (right-twisted) 0.0
Parallel 0.0
Turn 0.0
Others 10.4
10.4%
17%
72.7%
Helix1
Helix2
Anti1
Anti2
Anti3
Parallel
Turn
Others
Fitting results
Delta epsilon (M^-1 cm ^-1)
Wavelength (nm)
Experimental
Fitted
BESTSEL
RMSD 1.5959
NRMSD 0.0337
Comments
Input in text window.
CD units are converted to delta epsilon.
Maximum usable wavelength range: 190-250 nm.

(E1)

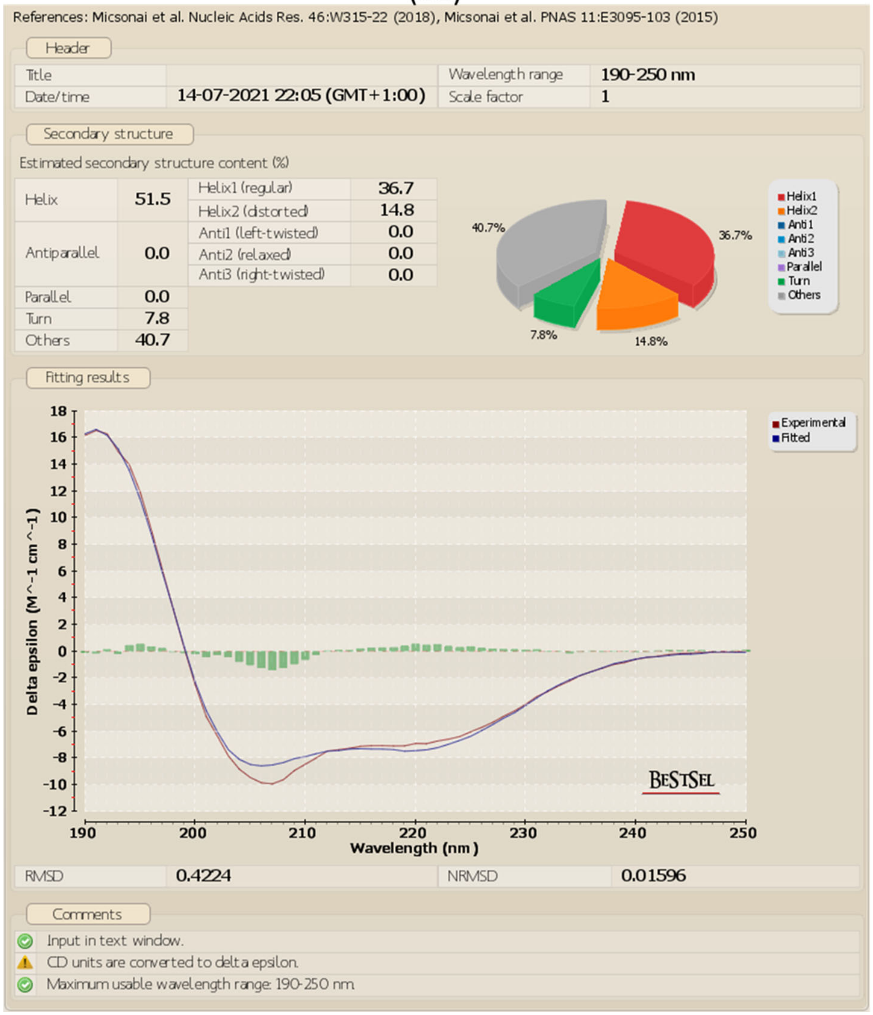
References: Micsonai et al. Nucleic Acids Res. 46:W315-22 (2018), Micsonai et al. PNAS 11:E3095-103 (2015)
Header
Title
Date/time
14-07-2021 22:05 (GMT+1:00)
Wavelength range
190-250 nm
Scale factor
1
Secondary structure
Estimated secondary structure content (%)
Helix 51.5
Helix1 (regular) 36.7
Helix2 (distorted) 14.8
Antiparallel 0.0
Anti1 (left-twisted) 0.0
Anti2 (relaxed) 0.0
Anti3 (right-twisted) 0.0
Parallel 0.0
Turn 7.8
Others 40.7
40.7%
36.7%
7.8%
14.8%
Helix1
Helix2
Anti1
Anti2
Anti3
Parallel
Turn
Others
Fitting results
Experimental
Fitted
Delta epsilon (M^-1 cm^-1)
Wavelength (nm)
BeStSel
RMSD 0.4224
NRMSD 0.01596
Comments
Input in text window.
CD units are converted to delta epsilon.
Maximum usable wavelength range: 190-250 nm.

(E2)

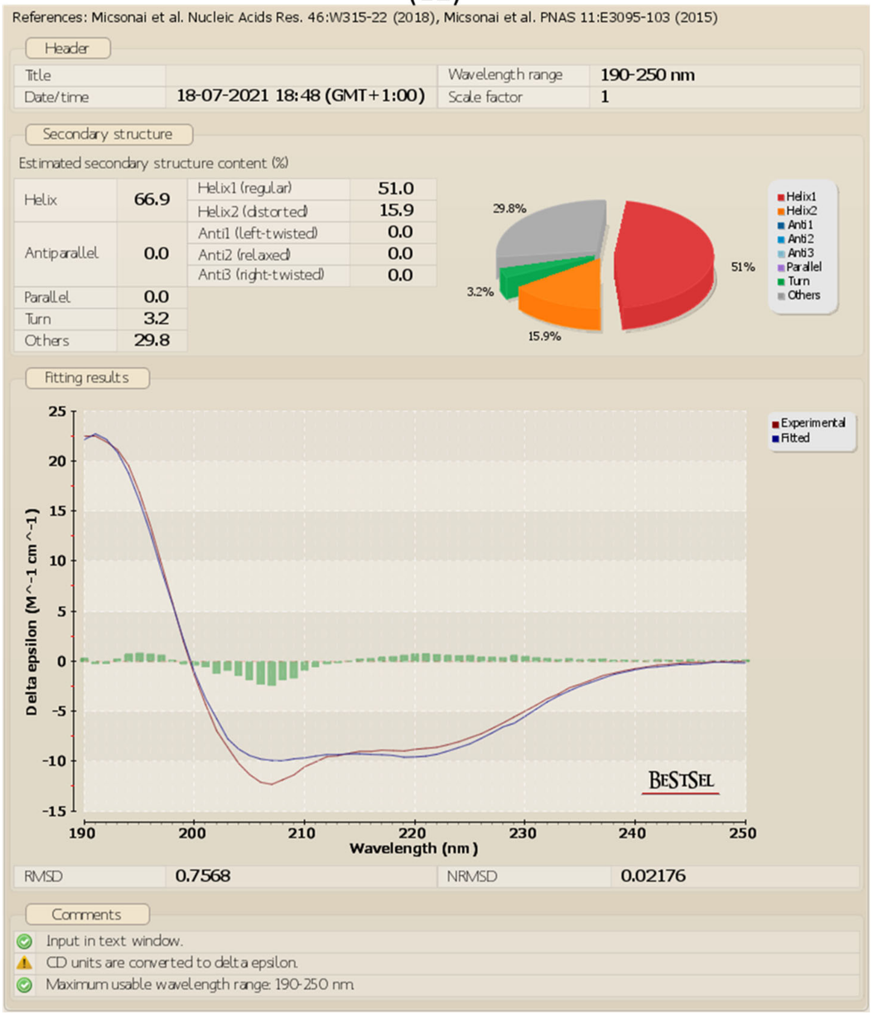
References: Micsonai et al. Nucleic Acids Res. 46:W315-22 (2018), Micsonai et al. PNAS 11:E3095-103 (2015)
Header
Title
Date/time
18-07-2021 18:48 (GMT+1:00)
Wavelength range
190-250 nm
Scale factor
1
Secondary structure
Estimated secondary structure content (%)
Helix 66.9
Helix1 (regular) 51.0
Helix2 (distorted) 15.9
Antiparallel 0.0
Anti1 (left-twisted) 0.0
Anti2 (relaxed) 0.0
Anti3 (right-twisted) 0.0
Parallel 0.0
Turn 3.2
Others 29.8
29.8%
51%
3.2%
15.9%
Helix1
Helix2
Anti1
Anti2
Anti3
Parallel
Turn
Others
Fitting results
Experimental
Fitted
Delta epsilon (M^-1 cm^-1)
Wavelength (nm)
BeStSel
RMSD 0.7568
NRMSD 0.02176
Comments
Input in text window.
CD units are converted to delta epsilon.
Maximum usable wavelength range: 190-250 nm.

(F1)

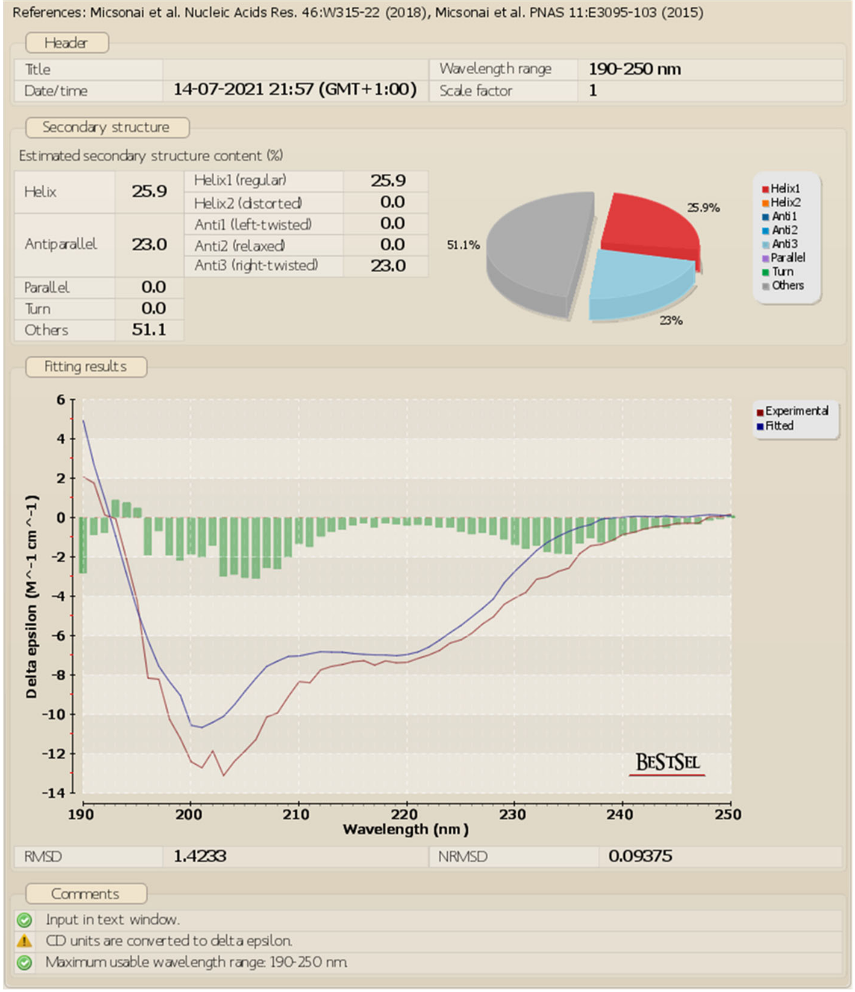
References: Micsonai et al. Nucleic Acids Res. 46:W315-22 (2018), Micsonai et al. PNAS 11:E3095-103 (2015)
Header
Title
Wavelength range 190-250 nm
Date/time 14-07-2021 21:57 (GMT+1:00)
Scale factor 1
Secondary structure
Estimated secondary structure content (%)
Helix 25.9
Helix1 (regular) 25.9
Helix2 (distorted) 0.0
Antiparallel 23.0
Anti1 (left-twisted) 0.0
Anti2 (relaxed) 0.0
Anti3 (right-twisted) 23.0
Parallel 0.0
Turn 0.0
Others 51.1
25.9%
51.1%
23%
Helix1
Helix2
Anti1
Anti2
Anti3
Parallel
Turn
Others
Fitting results
Experimental
Fitted
Delta epsilon (M^-1 cm^-1)
Wavelength (nm)
BeStSel
RMSD 1.4233
NRMSD 0.09375
Comments
Input in text window.
CD units are converted to delta epsilon.
Maximum usable wavelength range: 190-250 nm.

(F2)

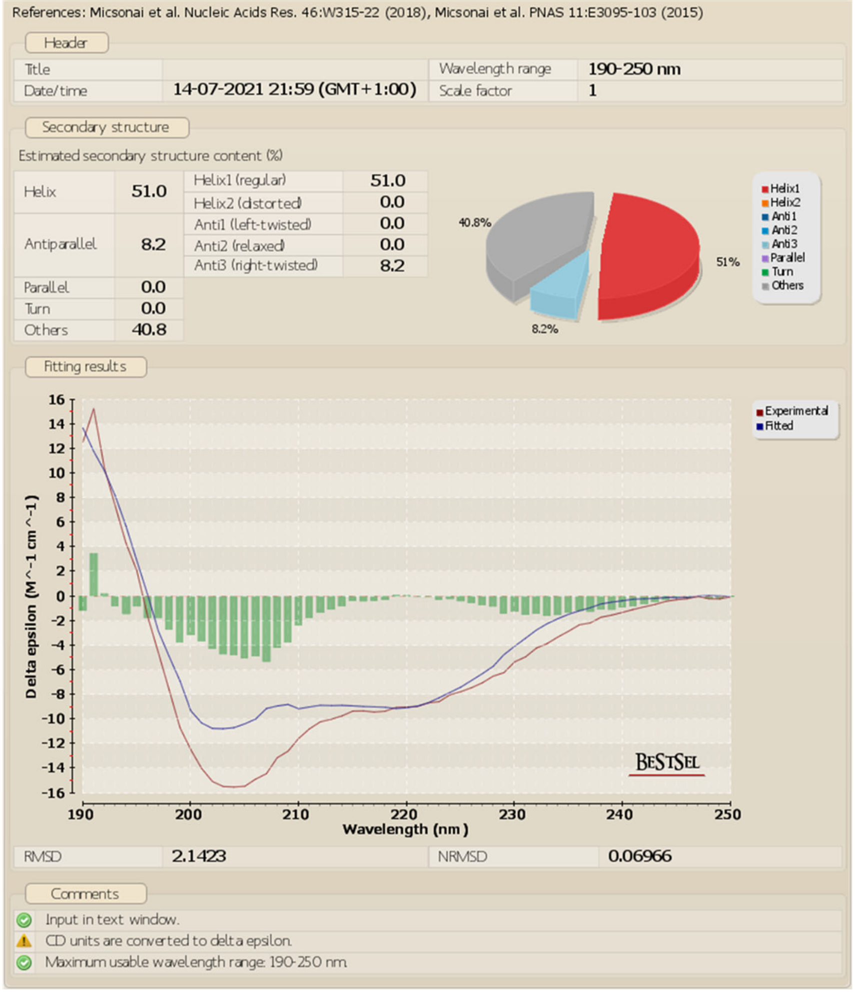
References: Micsonai et al. Nucleic Acids Res. 46:W315-22 (2018), Micsonai et al. PNAS 11:E3095-103 (2015)
Header
Title
Wavelength range 190-250 nm
Date/time 14-07-2021 21:59 (GMT+1:00)
Scale factor 1
Secondary structure
Estimated secondary structure content (%)
Helix 51.0
Helix1 (regular) 51.0
Helix2 (distorted) 0.0
Antiparallel 8.2
Anti1 (left-twisted) 0.0
Anti2 (relaxed) 0.0
Anti3 (right-twisted) 8.2
Parallel 0.0
Turn 0.0
Others 40.8
40.8%
51%
8.2%
Helix1
Helix2
Anti1
Anti2
Anti3
Parallel
Turn
Others
Fitting results
Experimental
Fitted
Delta epsilon (M^-1 cm^-1)
Wavelength (nm)
BeStSel
RMSD 2.1423
NRMSD 0.06966
Comments
Input in text window.
CD units are converted to delta epsilon.
Maximum usable wavelength range: 190-250 nm.

(G1)

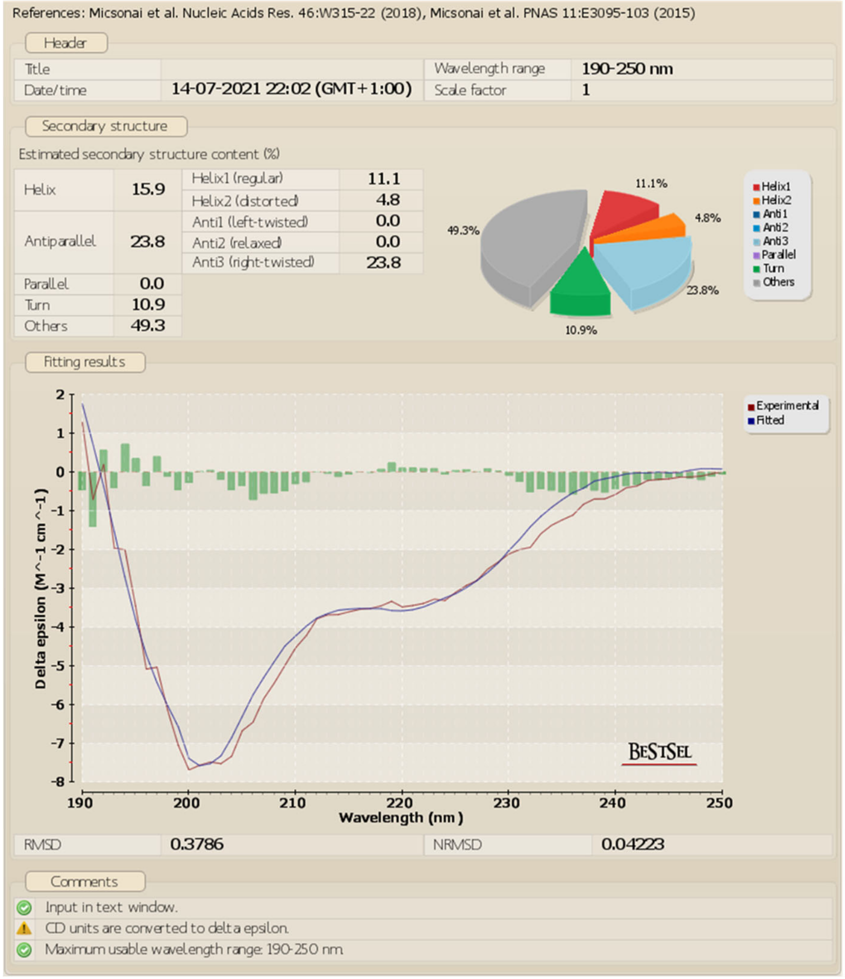
References: Micsonai et al. Nucleic Acids Res. 46:W315-22 (2018), Micsonai et al. PNAS 11:E3095-103 (2015)
Header
Title
Date/time
14-07-2021 22:02 (GMT+1:00)
Wavelength range
190-250 nm
Scale factor
1
Secondary structure
Estimated secondary structure content (%)
Helix
15.9
Helix1 (regular)
11.1
Helix2 (distorted)
4.8
Antiparallel
23.8
Anti1 (left-twisted)
0.0
Anti2 (relaxed)
0.0
Anti3 (right-twisted)
23.8
Parallel
0.0
Turn
10.9
Others
49.3
11.1%
4.8%
49.3%
23.8%
10.9%
Helix1
Helix2
Anti1
Anti2
Anti3
Parallel
Turn
Others
Fitting results
Experimental
Fitted
Delta epsilon (M^-1 cm^-1)
Wavelength (nm)
BeStSel
RMSD
0.3786
NRMSD
0.04223
Comments
Input in text window.
CD units are converted to delta epsilon.
Maximum usable wavelength range: 190-250 nm.

(G2)

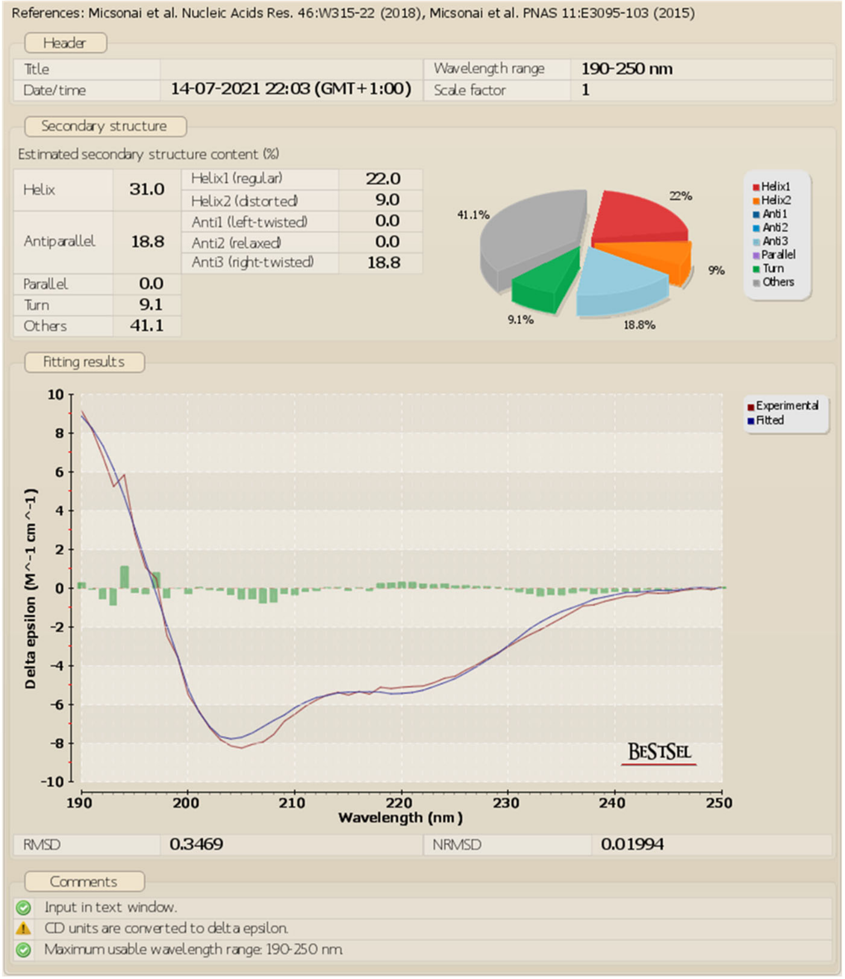
References: Micsonai et al. Nucleic Acids Res. 46:W315-22 (2018), Micsonai et al. PNAS 11:E3095-103 (2015)
Header
Title
Date/time
14-07-2021 22:03 (GMT+1:00)
Wavelength range
190-250 nm
Scale factor
1
Secondary structure
Estimated secondary structure content (%)
Helix
31.0
Helix1 (regular)
22.0
Helix2 (distorted)
9.0
Antiparallel
18.8
Anti1 (left-twisted)
0.0
Anti2 (relaxed)
0.0
Anti3 (right-twisted)
18.8
Parallel
0.0
Turn
9.1
Others
41.1
22%
41.1%
9%
9.1%
18.8%
Helix1
Helix2
Anti1
Anti2
Anti3
Parallel
Turn
Others
Fitting results
Experimental
Fitted
Delta epsilon (M^-1 cm^-1)
Wavelength (nm)
BeStSel
RMSD
0.3469
NRMSD
0.01994
Comments
Input in text window.
CD units are converted to delta epsilon.
Maximum usable wavelength range: 190-250 nm.

Figure S5. The estimated secondary structure contents analysed by Bestsel. The (A1) Brevinin-2GUb, (B1) tB2U, (C1) tB2U-α, (D1) tB2U-K, (E1) tB2U-6K, (F1) 7-tB2U-K and (G1) 14-tB2U-K was in the environment of 20mM NH_4Ac. The (A2) Brevinin-2GUb, (B2) tB2U, (C2) tB2U-α, (D2) tB2U-K, (E2) tB2U-6K, (F2) 7-tB2U-K and (G2) 14-tB2U-K was in the environment of 50% TFE in 20mM NH_4Ac.

Table S1. Two-way ANOVA analysis of haemolysis activity of Brevinin-2GUb and its analogues. The significance was presented by the symbol **** (P<0.0001).

Dunnett's multiple comparison test				
Peptide name	Groups	Mean Diff.	95% CI of diff.	Significance
	N vs. P	-100.7	-102.8 to -98.56	****
Brevinin-2GUb	N vs. 512 μM	-21.77	-24.01 to -19.53	****
	N vs. 256 μM	-8.063	-10.30 to -5.825	****
	N vs. 128 μM	-5.500	-7.739 to -3.261	****
tB2U	N vs. 512 μM	-3.363	-5.241 to -1.486	****
tB2U-α	N vs. 512 μM	-4.657	-6.534 to -2.779	****

tB2U-K	N vs. 512 µM	-11.94	-13.47 to -10.41	****
	N vs. 256 µM	-7.883	-9.328 to -6.438	****
	N vs. 128 µM	-6.718	-8.163 to -5.273	****
7-tB2U-K	N vs. 512 µM	-3.697	-5.864 to -1.529	****
14-tB2U-K	N vs. 512 µM	-4.780	-6.948 to -2.612	****
tB2U-6K	N vs. 512 µM	-6.963	-9.202 to -4.725	****
	N vs. 256 µM	-5.481	-6.926 to -4.036	****
	N vs. 128 µM	-4.051	-5.496 to -2.606	****

Table S2. Two-way ANOVA analysis of MTT assay of Brevinin-2GUb and its analogues against HaCaT. The significance was presented by the symbol **** ($P<0.0001$).

Dunnett's multiple comparison test				
Peptide name	Groups	Mean Diff.	95% CI of diff.	Significance
	N vs. P	99.69	97.42 to 102.0	****
Brevinin-2GUb	N vs. 10^{-4} M	54.35	52.08 to 56.63	****

Table S3. The survival analysis of the antimicrobial activities in vivo of Brevinin-2GUb and its analogues. The significance was presented by the symbol * ($P<0.05$), ** ($P<0.01$), *** ($P<0.001$) and **** ($P<0.0001$).

Log-rank (Mantel-Cox) Test				
Peptide	Groups	Chi Square	P value	P value summary
	N vs. P	19.26	<0.0001	****

Brevinin-2GUb	N vs. 30 mg/kg	15.01	0.0001	***
	N vs. 15 mg/kg	6.565	0.0104	*
	N vs. 7.5 mg/kg	6.350	0.0117	*
tB2U-K	N vs. 30 mg/kg	15.01	0.0001	***
	N vs. 15 mg/kg	8.308	0.0039	**
	N vs. 7.5 mg/kg	5.880	0.153	*
tB2U-6K	N vs. 30 mg/kg	19.26	<0.0001	****
	N vs. 15 mg/kg	13.95	0.0002	***
	N vs. 7.5 mg/kg	5.026	0.0250	*
